# An Ethical Assessment Tool (ETHAS) to Evaluate the Application of Assisted Reproductive Technologies in Mammals’ Conservation: The Case of the Northern White Rhinoceros (*Ceratotherium simum cottoni*)

**DOI:** 10.3390/ani11020312

**Published:** 2021-01-26

**Authors:** Barbara de Mori, Maria Michela Spiriti, Ilaria Pollastri, Simona Normando, Pierfrancesco Biasetti, Daniela Florio, Francesco Andreucci, Silvia Colleoni, Cesare Galli, Frank Göritz, Robert Hermes, Susanne Holtze, Giovanna Lazzari, Steven Seet, Jan Zwilling, Jan Stejskal, Samuel Mutisya, David Ndeereh, Stephen Ngulu, Richard Vigne, Thomas B. Hildebrandt

**Affiliations:** 1 Department of Comparative Biomedicine and Food Science, University of Padua, 35020 Padua, Italy; mariamichela.spiriti@studenti.unipd.it (M.M.S.); ilaria.pollastri@studenti.unipd.it (I.P.); 2 Ethics Laboratory for Veterinary Medicine, Conservation and Animal Welfare, University of Padua, 35020 Padua, Italy; biasetti@izw-berlin.de (P.B.); daniela.florio@unibo.it (D.F.); francesco.andreucci@aulss5.veneto.it (F.A.); 3 Department of Reproduction Management, Leibniz Institute for Zoo and Wildlife Research, D-10315 Berlin, Germany; goeritz@izw-berlin.de (F.G.); hermes@izw-berlin.de (R.H.); holtze@izw-berlin.de (S.H.); 4 Department of Veterinary Medical Science, University of Bologna, 40064 Bologna, Italy; 5 Avantea, Laboratory of Reproductive Technologies, 26100 Cremona, Italy; silviacolleoni@avantea.it (S.C.); cesaregalli@avantea.it (C.G.); giovannalazzari@avantea.it (G.L.); 6 Avantea Foundation, 26100 Cremona, Italy; 7 Science Communication, Science Management, Leibniz Institute for Zoo and Wildlife Research, D-10315 Berlin, Germany; seet@izw-berlin.de (S.S.); zwilling@izw-berlin.de (J.Z.); 8 ZOO Dvůr Králové, 54401 Dvůr Králové nad Labem, Czech Republic; jan.stejskal@zoodvurkralove.cz; 9 Ol Pejeta Wildlife Conservancy, Nanyuki 10400, Kenya; samuel.mutisya@olpejetaconservancy.org (S.M.); stephen.ngulu@olpejetaconservancy.org (S.N.); richard.vigne@olpejetaconservancy.org (R.V.); 10 Kenya Wildlife Service, Nairobi 00100, Kenya; dndeereh@kws.go.ke; 11 Faculty of Veterinary Medicine, Free University of Berlin, D-14195 Berlin, Germany

**Keywords:** northern white rhinoceros, assisted reproductive technologies, ethical self-assessment, conservation ethics, animal welfare, ethical risk assessment, ethical review process

## Abstract

**Simple Summary:**

Applying assisted reproductive technologies (ARTs) to the conservation of endangered species may be the only way to save them from extinction. However, ART application can raise relevant ethical issues and could benefit from a comprehensive ethical assessment. Unfortunately, there is a lack of attention to the topic in the scientific literature and, to our knowledge, there is no tool for the ethical assessment of ARTs in the context of conservation that has been described. In the present paper, we show the effects of applying a dedicated ethical self-assessment tool, the Ethical Assessment Tool (ETHAS), to ovum pick-up and in vitro fertilization procedures performed within the B*io*R*escue* project. The B*io*R*escue* project is an international enterprise using ARTs to save the northern white rhinoceros from extinction. The situation of the northern white rhinoceros is particularly critical as there are only two individuals of this subspecies still alive and they are both infertile females. The application of the ETHAS to the procedures contributed to the overall acceptability of the project and improved communication among the project’s partners. In turn, the tool itself was also refined through an iterative consultation process between experts (both ethicists and scientists) and stakeholders.

**Abstract:**

Assisted reproductive technologies (ARTs) can make a difference in biodiversity conservation. Their application, however, can create risks and raise ethical issues that need addressing. Unfortunately, there is a lack of attention to the topic in the scientific literature and, to our knowledge, there is no tool for the ethical assessment of ARTs in the context of conservation that has been described. This paper reports the first applications of the Ethical Assessment Tool (ETHAS) to trans-rectal ovum pick-up (OPU) and in vitro fertilization (IVF) procedures used in a northern white rhinoceros (*Ceratotherium simum cottoni*) conservation project. The ETHAS consists of two checklists, the Ethical Evaluation Sheet and the Ethical Risk Assessment, and is specifically customized for each ART procedure. It provides an integrated, multilevel and standardized self-assessment of the procedure under scrutiny, generating an ethical acceptability ranking (totally, partially, not acceptable) and a risk rank (low, medium, high), and, hence, allows for implementing measures to address or manage issues beforehand. The application of the ETHAS to the procedures performed on the northern white rhinoceros was effective in ensuring a high standard of procedures, contributing to the acceptability and improved communication among the project’s partners. In turn, the tool itself was also refined through an iterative consultation process between experts and stakeholders.

## 1. Introduction

In the present global scenario, where an accelerated rate of extinction is paired with a severe decline in populations’ abundance in surviving species [1,2], assisted reproductive technologies (ARTs) can make a difference in biodiversity conservation. ARTs can raise the chance of success of conservation breeding programs by both overcoming infertility issues and optimizing genetic management, avoiding inbreeding (or outbreeding) depression and risks of transmission of inherited diseases [3,4,5].

ARTs, in fact, may offer the only chance for survival of many endangered species with very fragmented populations or only few extant individuals. In this case, ARTs can be employed not only to boost the number of offspring, but also to enhance the genetic exchange between the fragmented populations (living both in situ and ex situ) without the need of actually translocating the animals [6], ARTs can also enhance the genetic exchange between living and dead generations by using gametes stored in cryobanks [7] or, in what could be a possible near-future development of this biotechnology, produced from stem cells [8].

While ARTs are a robust opportunity in the conservationist’s toolbox—and one which promises to become increasingly important in the future—their application may raise several ethical issues. The use of ARTs can raise ethical concerns also in human medicine, some of which can be still valid when ARTs are applied to non-human animals, but many of the issues raised by the application of these technologies in conservation breeding projects are more specific [9,10,11]. These may range from issues also common in applications of ARTs to livestock [12,13,14] to more specific issues tied to the particular context of biodiversity conservation. For instance, ARTs need species-specific optimization in order to be successfully employed, and this, in turn, depends on detailed knowledge of the reproductive biology of the species involved [4]. Such knowledge may be difficult to obtain in already endangered species, due to the limited numbers of available individuals for research and the potential difficulties in accessing them [15]. In the end, its pursuit may pose several dilemmas to scientists and conservationists intentioned to both safeguard the remaining individuals of a species and obtain enough information for a last attempt to reverse its decline. It could also be claimed that important resources—in terms of time, space, people, competencies, and funding [16], which are needed to implement conservation projects involving ARTs, from the first step of species-specific optimization of the techniques to the breeding and reintroduction steps—could be perhaps better allocated to other more traditional forms of biodiversity conservation. Moreover, from a more theoretical perspective, applying ARTs could be seen as an exemplary case of “technofix” [11,17], that is, the short-sighted use of technology as a way to sort out the outcome of morally problematic activities instead of addressing their causes, or as an apparently “easy” solution to the decline in wildlife populations, with the risk of inducing complacency in the problem.

Above all, a crucial source of ethical concern regarding ARTs in biodiversity conservation is animal welfare. Many applications of ARTs require manipulation of live animals and, in some case, invasive procedures, with real risks for their welfare. This is of course also true for farm animals, where the issue has not received enough attention (for instance, [18,19]), but is further exacerbated in wildlife, where at least three factors intervene to complicate the matter. The first is the experimental characters of many ARTs applications to wildlife, with procedures less established than in livestock and which often stand in a gray area between research and veterinary practice. The second is our knowledge on animal welfare science, which, again, is scarcer in wildlife than in farm or laboratory animals. The third concerns manipulation of the animals. While livestock and, in general, domestic animals are more accustomed to being manipulated by people, operating on wildlife may be more stressful for the animals involved (and also for the staff performing the procedures) and may be more demanding in terms of restraint, sedation, or anesthesia. Moreover, this higher toll exacted in terms of animal welfare may be more difficult to mitigate, since excessive conditioning of the animals involved in the procedures could be undesirable due to the need for minimizing the effects of captivity [20].

In general, when an ethical assessment of a procedure involving individual animals has to be carried out, the golden standard would be a systematic project evaluation, requiring, among other things: (i) a risk assessment; (ii) an assessment of welfare conditions and pain, suffering, distress, and lasting harm imposed on the animals; (iii) a harm–benefit evaluation; and (iv) the application of the 3Rs (Replacement, Reduction and Refinement) [21]. This standard is, at least in theory, systematically applied when research projects involving laboratory animals are submitted to ethical committees for evaluation. With regard to wildlife, however, this standard evaluation is not performed systematically. Yet this evaluation is crucial, especially for projects involving ARTs.

Risk assessment, for instance, should be considered essential in these cases. Application of ARTs to wildlife and their biomaterial entails accepting a certain grade of uncertainty. This requires a prior definition of the ethically tolerable risk threshold for the procedures, which can be conducted only by performing a detailed risk analysis, based on traditional risk analysis [22], specific animal welfare [23] and ethical risk analysis [24], and application of the precautionary principle [25,26,27,28].

The assessment of potential pain, suffering, distress, and harm, alongside general welfare conditions of the individual animals involved in the procedure, should also be considered essential. However, pain, suffering, distress, harm, and, in general, the welfare of the individual animals have traditionally played a secondary role in biodiversity conservation. This is partly due to the fact that the goals of biodiversity conservation and of animal welfare are conceptually distinct and may sometimes diverge, since the former is mainly focused on species, whereas the latter is focused on individuals [29,30,31]. Nevertheless, excessive divergence may remove societal support for conservation projects [32,33]. Moreover, animal welfare is a crucial factor in the success of conservation breeding and reintroduction programs [20,34]. Yet, as already noted, the assessment of wildlife welfare may be harder to obtain. Knowledge on the issue is lacking if compared to laboratory animals. This is both due to fewer research works on the former subject than on the latter and to the larger number and diversity of wild vertebrate species compared to the few taxa employed in laboratory research [35]. These difficulties, however, do not remove the need to carefully assess the general welfare conditions and the specific potential pain, suffering, distress and lasting harm imposed on the animals during the application of the ART procedures.

The third important requirement is harm–benefit analysis. Again, while this is nowadays routine in the ethical assessment of laboratory projects involving animals [36], it is instead underrepresented in wildlife studies. In particular, harm–benefit analysis has been rarely applied to evaluate the impact on the health and welfare of wild animals involved in veterinary procedures aimed at safeguarding their species [37]. Nonetheless, it is progressively used to identify costs and benefits arising from conservation projects in relation to not only their economic impact [38], but also to their positive or negative consequences for the ecosystem and the local wildlife population.

The same can be said also for the fourth requirement, the application of the 3Rs, which has been widely satisfied in laboratory research but rarely in wildlife studies, where research conditions are more heterogeneous and it is harder to standardize a methodology for its implementation as has been done in laboratory research. However, as progressively stated [39,40], the 3Rs principle is crucial also for wildlife research. For instance, replacement can be obtained with non-invasive research techniques, reduction with optimized experimental design and refinement with better methods of capture, anesthesia and handling [39].

It may be countered that conservation interventions do not qualify—at least in a full sense—as research and, as such, should not be subjected to the same stringent standards involved in laboratory research. However, as already noted, the boundaries between research and veterinary practice are often blurred when applying ARTs to conservation breeding programs. Moreover, most applications of ARTs to wildlife may take place both in research and non-research scenarios. This raises a boundary problem, as the same activity may be subjected to different ethical standards of evaluation when performed in different contexts. To solve this inconsistency, it has been suggested that far from relaxing our ethical standards on research, we should instead extend them to all similar activities [41,42].

For all these reasons, conservation projects incorporating ARTs should be carefully scrutinized in order to evaluate their ethical acceptability, using the highest procedural standards and compliance with best practices and regulations as landmarks. Currently, despite the increasing interest in the use of ARTs in conservation, there is little attention to ethical assessment and, to our knowledge, there are no tools to evaluate the specific risks and ethical aspects involved. A simple search on Scopus (https://www.scopus.com/), with “ethical assessment” AND “reproduction” and “wild” and “animal” as keywords run in December 2020, gave no results. One of the reasons for this result could be that, with ARTs being applied to conservation breeding projects often in the gray area between clinical practice and research, their use in such context often does not require external ethical approval. It is therefore even more important that the practitioners and the researchers involved in these types of projects are able to evaluate the potential ethical relevant issues spanning from the procedures they use themselves. One way to enable practitioners and researchers to evaluate their procedures is to provide them with a comprehensive and customizable tool for the self-assessment of such procedures, which, once developed by experts with an ethics background (specifically, in applied ethics related to conservation and animal welfare), can be used also by people lacking such background. Self-assessment could also be an important step in preparation for an external overall evaluation of the ethical acceptability of a project and could help scientists to be proactive and to scrutinize the ethical issues surrounding their work [43].

In this paper, we present the self-ethical assessment of two ART procedures performed in the context of a conservation breeding program aimed at avoiding the extinction of the northern white rhinoceros (*Ceratotherium simum cottoni*—NWR). The procedures involved both southern white rhinoceros (SWR) females in European zoos and the last two surviving NWRs. The assessment was preformed using a self-assessment tool explicitly designed for conservation breeding programs, the Ethical Assessment Tool (ETHAS), as customized for the self-assessment of ovum pick-up (OPU) and in vitro fertilization (IVF) procedures. The aim of the study was to investigate both whether applying the tool could contribute to ensuring a high standard and improvement of procedures being assessed and, at the same time, how applying the first version of the tool in actual field conditions contributes to shape and improve the tool itself.

## 2. Materials and Methods

### 2.1. The Case

The NWR, a subspecies of the white rhinoceros (*Ceratotherium simum*), once ranged over much of the savannah of Central Africa [44,45,46]. However, between the 1970s and the 1980s, the wild population was reduced to only 15 individuals, and there have been no reported signs of their presence in the wild since 2007. Nowadays, it is declared as “possibly extinct in the wild” [47], as the only remaining individuals live in captivity. The last remaining individuals are two females, Najin and Fatu, who are under constant surveillance at Ol Pejeta Conservancy, in Kenya, and cannot have a viable pregnancy due to health and age-related issues. Najin is 31 years old and has a large ovarian tumor on her left adnexus. Moreover, she has very weak hind legs due to bilateral alterations of the Achilles tendons. Her 20-year-old daughter Fatu has developed untreatable degenerative endometriosis of unknown cause over her entire uterus [48]. Therefore, the only chance to save this iconic subspecies from the brink of extinction is to utilize ART procedures, using in vitro embryos gestated by recipient mothers of the sister subspecies—the southern white rhinoceros (*Ceratotherium simum simum*—SWR). In order to produce embryos, however, gametes have to be obtained first. During the last two decades, scientists have collected the semen from four NWR bulls and cryopreserved it in three different cryobanks [48]. No oocytes, instead, have ever been stored because of their low permeability to cryoprotectants and consequent susceptibility to chilling [49]. This means that ovum pick-up (OPU) has to be repeatedly performed on the two surviving females, in order to obtain viable oocytes, which are then sent to a specialized laboratory for incubating, maturing and performing in vitro fertilization (IVF), in order to obtain viable embryos. The embryos are then stored in liquid nitrogen, until transferred into an SWR recipient mother. OPU on Najin and Fatu was performed for the first time on August 22th, 2019, in collaboration with the Kenya Wildlife Service (KWS), and has been repeated three more times. Despite the difficulties caused by the COVID-19 pandemic, at present, there are five embryos ready for transfer.

When conducting an ethical assessment on ART procedures involving Najin and Fatu, the health-related issues of the two individuals are likely to be very relevant both because, as already said, they prevent the two animals from having a viable pregnancy and they impact on their welfare, mainly by modifying the risks that ART procedures create for the involved animals. In rhinoceroses, in general, OPU needs full anesthesia [50,51], with the animal lying down, and thus it may be a risky procedure even in healthy animals [48,52,53]. The scientific literature and best practices show that rhinoceroses quickly recover from ovum pick-up [54,55]—as fast as farm animals—making repeated anesthesia possible even within a short time period [51,56,57]. The health situation of the two NWRs may alter the risks posed by repeated anesthesia because their chronically ill status might affect their resilience to the procedure. However, the fact that they suffer from health issues increases the importance of being able to perform OPU with a higher frequency on them, in order to have more chances to succeed in saving the species from utter extinction, since their health issues might adversely affect their life expectancy and thus the time available for scheduling OPU.

Given the complexity of the ethically relevant issues involved, a sub-project dedicated to the development of a specific ethical self-assessment tool which could be used in mammalian conservation breeding programs was created within the B*io*R*escue* project—the international consortium led by the Leibniz Institute for Zoo and Wildlife Research of Berlin (Leibniz-IZW) and comprising the Czech Dvůr Králové Zoo, Avantea laboratory, Max Delbrück Center for Molecular Medicine (MDC), Kyushu University and Padua University (and having the support of other international partners), which is in charge of the whole project that involves Najin and Fatu and aims at avoiding the final extinction of the northern white rhinoceros.

### 2.2. The Tool (ETHAS)

The Ethical Assessment Tool (ETHAS) is a flexible and customizable instrument for the ethical self-evaluation of specific ART procedures applied to mammals in biodiversity conservation projects. It includes and integrates with each other risk assessment (general, ethical and welfare), pain/distress/welfare evaluation, harm–benefit analysis and the 3Rs tenet application. As already stated, self-assessment tools help scientists to be proactive and to scrutinize the ethical issues surrounding their work and are preliminary for an external overall evaluation of the ethical acceptability of a project [43]. Their implementation fosters dialogue between all participants and may lead to the actual improvement of the procedures. Moreover, routinely performed ethical self-assessment helps scientists to comply with ethical principles, best practices with animals, relevant legislation and authorizations and ethical approval [35]. Self-assessment cannot replace ethical assessment by an external committee, but it contributes both to the final acceptance of the project, by anticipating its possible ethically critical issues (and hence allowing for timely and comprehensive design of mitigation strategies), and to the communication of its results to the general public.

ETHAS is based on checklists, a tool commonly used in medicine and other fields to identify errors, ameliorate operational standards and comply with best practices [58,59]. Checklists are a valuable tool for self-assessment. Their use improves research results and makes them easier to be communicated, contributing to the responsible conduct of research, thereby increasing its public acceptance [35,43]. Moreover, they can be used by both experienced and inexperienced personnel alike, and they are easily understandable and verifiable [59].

ETHAS’s checklists aim to combine risk assessment with ethical acceptability assessment. Risk assessment is a crucial phase of risk analysis, and therefore it is very important for the overall ethical acceptability of wildlife conservation projects. As it is known, risk analysis is a three-step process: (i) risk evaluation/assessment, (ii) risk management and (iii) risk communication [60,61]. It allows a standardized, repeatable, transparent and documented evaluation of the risks posed by a course of action or a chain of decisions [62]. The use of ARTs on wild animals entails the acceptance of a certain level of risk, but this level must conform to the “as low as reasonably applicable principle” (ALARP) [63]. 

Therefore, the general frame of the ETHAS tool is based on two integrated checklists for self-assessment, the Ethical Evaluation Sheet (EES) and the Ethical Risk Assessment (ERA). Each ERA item is conceptually linked to a corresponding part of the EES checklist, which comprises, among others, all the relevant ethical aspects that are investigated in ERA. The link is reported in a column with an alphanumeric code.

There are customized EES and ERA versions for each ART procedure, but all share some common features. These constituent checklists of both EES and ERA have been developed on the basis of the current literature and best practices guidelines and refined through an iterative consultation process between experts (both ethicists and scientists) and stakeholders, which is still ongoing in the present stage of the project. They merge risk analysis, based on a combination of traditional, animal welfare and specific ethical risk assessments, with ethical analysis, based on pain/distress/welfare evaluation, harm–benefit analysis and the 3Rs tenet application, with the aim of defining the overall ethical acceptability of the procedure under assessment.

#### 2.2.1. Ethical Evaluation Sheet (EES)

The Ethical Evaluation Sheet (EES) highlights potential ethical issues arising from the ART application. As with corresponding tools for the ethical assessment of research projects with laboratory animals [43,64,65,66], the general frame of EES consists of four main sections of investigation: (a) Documents; (b) Harm–benefit evaluation; (c) Procedure quality evaluation; and (d) Scientific team quality evaluation. For each specific ART procedure, it is necessary to detail a certain number of items within these main sections. In the first trial, the EES for the OPU procedure consisted of a total number of 83 items, whereas the IVF-lab EES consisted of 64 items. However, since some items are made up of sub-items, the total possible answers counted in the final score can be more. Regarding the OPU EES, the total number was 88, while in the IVF-lab EES, it was 81. After the revision of some items, detailed in Section 3.2, a second version of both the OPU and the IVF-lab EES was developed. The second version of the EES for the OPU procedure consisted of a total number of 86 items, with a total number of 91 items and sub-items, whereas the second version of the IVF-lab EES had 66 items, with a total number of 83 items and sub-items.

Table 1 shows the general structure of the EES checklists for OPU and IVF procedures in more detail and reports the scientific sources of information used in their development.

The EES is designed to be filled in only once (unless the procedure’s protocol is changed) before to start the procedures. In the case of the procedures performed during the present study, as it was a phase in the development of the final version of the tool, the EES was filled in by a member of the B*io*R*escue* team with an ethical background in applied ethics in conservation and animal welfare. However, as underlined in the Introduction, in the final version of the tool, any member of the team performing the procedures will be able to fill in the EES, without the need of a specific ethical background. During the EES compilation, it is asked to answer “yes” or “no” to all items, depending on whether the requirements are met or not. Moreover, for some EES items, it is required to add further information to explain the answer. The EES is evaluated using a semi-quantitative scoring model in which the answers “yes” or “no” assume the value of 0 and 1, respectively. The sum of the items’ outcome divided into three homogeneous ranges defines the rank of the ethical acceptability of the procedure: not acceptable, partially acceptable, acceptable. Therefore, the final score obtained from the EES compilation identifies one of the three acceptability ranks. Table 2 describes the EES final score for the OPU and IVF procedures performed in the present study.

The identified acceptability level that represents the outcome of the EES assessment (defined as the first review level) defines the degree of the procedure acceptability. In case of a partial or not acceptable result in the ethical assessment, detected with the first review level, each section of the EES checklist is assessed individually. This second review level identifies at which section of the procedure corrective actions need to be planned. Finally, a third review level allows identifying the items whose requirement is not met and, therefore, the critical issues of the procedure to be reviewed before the procedure begins.

#### 2.2.2. Ethical Risk Assessment (ERA)

The ERA checklist is specifically customized for each procedure under scrutiny by identifying the appropriate phases for risk assessment. The scientific literature on ARTs has been revised to analyze, in detail, each step of the OPU and IVF procedures and detect possible hazards and ethical risks whose occurrence could negatively impact on the animal welfare, staff safety and procedure outcome [83]. As shown in Table 3, the OPU ERA is composed of five different phases: A) Identification of the individual/s, welfare assessment and procedure planning; B) Ovarian stimulation protocol; C) Anesthetic procedure for oocyte recovery; D) Oocyte recovery by transrectal procedure; and E) Gametes packaging. The total number of items in the OPU ERA first version was 52, while in the second, it was 56. Since some items are made up of sub-items, the total number of the first version was 91, while that of the second one was 101. Table 3 shows the OPU ERA checklist in more detail and reports the scientific sources of information used in its development.

The IVF-lab ERA, instead, as shown in Table 4, is composed of nine phases: (A) Laboratory quality assessment and specimens processing; (B) Gametes shipping to the laboratory; (C) Gametes biobanking; (D) Gametes preparation for ICSI; (E) Intracytoplasmic sperm injection (ICSI); (F) Embryos culture; (G) Embryos cryopreservation and biobanking; (H) Embryos packaging; and (I) Embryos shipping. The total number of items in the IVF-lab ERA was 72. Since some items are made up of sub-items, the total number was 103.

Each item and sub-item of the ERA checklists analyzes an element of the procedural step which could cause a hazard to the success of the phase under assessment. For each item, it is required to record a “yes” or “no” whether the requirement of the item is satisfied or not. Depending on the characteristics of the requirement and on the severity of the consequences associated with the hazard scenario, each item is scored differently (Table 5). For example, the consequences associated with a failure highlighted with items in phases A, B, C and D of the OPU ERA have different effects. Non-compliance with operational or animal management requirements has a more significant impact on animal welfare than non-compliance with operational instructions or documentary, structural, instrumental and environmental requirements (Table 5). The items of phase E of the OPU ERA have been evaluated with the risk categories of the IVF-lab ERA due to the consequences of the hazard impact on the gametes’ safety. In the IVF-lab ERA, three scoring ranges were defined on the basis of the type and severity of the possible outcomes that the hazard scenarios could have on gametes and embryos.

The assessment uses a semi-quantitative scoring model where the risk is determined by a single value R that combines the probabilities (*p*) and consequences (x) associated with the occurrence of a hazard scenario [112]. The hazard scenario is identified with each ERA item. The probabilities are determined by the satisfaction or not of the item. The consequences depend on the characteristics of the requirement of the item and are classified into different levels of severity, in accordance with Table 5.

$$R\;=\;\overset{n}{\underset{i\;=\;1}{\sum }}p_{i}x_{i}$$

In the specific model, n corresponds to the number of scenarios chosen to describe the risk (number of items of the ERA checklist), *p_i_* can assume values of 0 or 1 depending on whether the requirement is met (yes) or not (no/no answer) and *x_i_* is from 1 to 3, as described in Table 5.

ERA checklists are designed to be filled in each time a procedure is performed. They have to be filled in by one to three different people, depending on the procedure under assessment, with two main aims: to have an overview of the procedure and to verify, in case of more persons involved in the assessment, if communication regarding ethically relevant issues among the participants is effective. Regarding the OPU procedure, for instance, if it is executed only by the veterinary staff of the zoo or facility hosting the animals, the ERA can be filled in just by the chief veterinarian. If the OPU procedure is executed by an external veterinary team, the ERA has to be filled in both by the external and internal veterinarians and the zoo or facility managing director. In the applications of the ETHAS described in the present paper, three different participants responded to the OPU ERA for both the procedures performed: the veterinarian responsible for the B*io*R*escue* project, the local veterinarian and the managing director of the facility where the procedure took place.

Regarding the second aim—to verify if communication is effective—the three answers for each item are entered in an Excel spreadsheet, and the modal value that allows highlighting the most frequent responses per set of answers is calculated. The sum of the modal values is divided into three ranges, identifying the three categories of risk severity (low, medium, high). On the contrary, the modal value is not necessary at all for the IVF-lab ERA because it is compiled by only one person—the person responsible for the IVF laboratory. In this case, the sum of the values of each answer is divided into three ranges, corresponding to the three risk categories (Table 6).

Similarly to the EES, also for the ERA, three review levels can be applied: at an overall level (risk rank, first review level), at the phase level (second review level) and at the items level (third review level). The review levels allow revising the specific application of the procedure in case of the detection of a medium or high risk rank and applying risk management and risk communication strategies.

#### 2.2.3. Final Overall Evaluation (EES + ERA)

The ETHAS generates a risk rank (low, medium, high) through the ERA and an ethical acceptability rank (totally, partially, not acceptable) with the EES. The overall final evaluation (ERA + EES) is calculated by combining the acceptability ranking obtained from the EES and the risk rank obtained the from ERA (Table 7). Therefore, ETHAS overall evaluation falls into three categories:

(1) Acceptable, when the ESS results in totally acceptable and the ERA detects low risks. The assessed procedure may be accepted without further actions.

(2) Acceptable with mitigation, when the EES results in partially acceptable and the ERA detects medium risks. The assessed procedure may be accepted only if critical issues are identified and addressed and the specific application of the procedure is revised.

(3) Not acceptable, when the EES detects a not acceptable result and the ERA detects high risks. The assessed procedure may be unacceptable until further improvements are enforced to eliminate the associated ethical concerns and procedural risks.

Scoring of both checklists and the overall final evaluation have to be performed by the person completing the EES.

After the risk assessment, the ETHAS enables risk management of the possible highlighted hazards. Risk management (the second phase of a risk analysis process) allows raising awareness of the potential hazards and risks and enables the sharing and acceptance of the measures to be adopted to reduce the risks. Risk mitigation actions have to be chosen taking into account: (1) the characteristics of the requirements (in terms of scoring); and (2) what is reasonable and technically possible. Moreover, risk management allows an exchange of information and opinions between the staff involved in the ART procedures.

Finally, the ETHAS enables also risk communication: through an iterative process among the staff directly involved in the procedures, information and opinions on hazards and their associated risks are exchanged, allowing a transparent and overarching discussion of results.

### 2.3. Application of the Tool

In a preliminary phase of ETHAS development, after consulting the relevant scientific literature and best practice guidelines on OPU and IVF procedures, a draft of the checklists was designed using a bottom-up approach, by witnessing several procedures and discussing with the teams performing them the main areas identified by the scientific literature and best practices. Relevant areas, not previously found in the literature search, but found to be relevant in the practical application of the ART procedures, were also added and discussed. The OPU procedures witnessed in the preliminary phase included both procedures performed on infertile SWRs in European zoos—who were involved in the B*io*R*escue* project both for approaching their infertility problems and for protocol optimization—and those (August 2019) performed on Najin and Fatu, in order to ensure suitable consideration of the relevant specific features of these individuals (e.g., their health status, as discussed in 1.1.) in the tool. The IVF procedures witnessed were all performed at the Avantea laboratory, which up to now is the only one that produced a viable rhinoceros embryo.

The preliminary phase led to the first version (beta1) of the ETHAS customization for OPU procedures (OPU EES + OPU ERA). The complete beta1 version can be found as Supplementary Material (File S1 and S2). The beta1 ETHAS version was then applied in October 2019 during an OPU procedure performed by the B*io*R*escue* team on three sub-fertile or infertile SWR females housed in a European zoo, in order to evaluate both the effects of conducting ethical self-assessment on the application of ART procedures and to improve the beta version of the tool itself.

The application of the beta1 version led to the revisions of some items, detailed in Section 3.2, resulting in the creation of an updated version (beta2) of the OPU EES and ERA. The beta2 version was applied in December 2019, during an OPU procedure performed by the B*io*R*escue* team on the last two NWRs in Kenya. Both procedures (October and December) were performed following the B*io*R*escue* team’s standardized protocols. Similarly, the first version (beta1) of the ETHAS customization for IVF procedures (IVF-lab EES + IVF-lab ERA) was first applied in August 2019 (Supplementary Material File S3 and S4), and the second one (beta2, after the changes detailed in Section 3.2) was applied in October 2019, at the Avantea laboratory.

## 3. Results

### 3.1. How Applying the Tool Contributed to the Refinement of The Procedures

#### 3.1.1. EES

In both the first and second assessment trials, the ethical assessment of OPU and IVF-lab resulted in “Totally acceptable” in both EESs (Table 8). However, despite this result, the EESs were investigated at the second and third review levels to examine whether there were unmet requirements and, if so, in which sections and items they were found.

The OPU EES in the first trial received a final score of 3 over 88, while in the second trial, it received a final score of 3 over 91. In both trials, the three negative answers were detected in the “Harm–benefit evaluation of the procedure” section. The first of the three unmet requirements was related to the fact that infertility is not widespread in the SWR wild population. For this reason, even if it is fundamental to optimize the procedure for this subspecies in zoos and facilities alike, there is no wilder population that can receive a direct benefit from this process. Nevertheless, the acquired knowledge on the rhinoceroses’ reproduction might turn out to be useful in the future, also for the other rhino species. The second concerns the possibility that the OPU procedure may have adverse side effects on the animal under it in case of a harmful event. Even if all the precautions are taken, the risk probability is never zero. Finally, the third one was related to the fact that any adverse event on the last two NWR females impacts this subspecies.

Regarding the IVF-lab EES first trial, the final score was 1 over 81, while the IVF-lab EES second trial obtained a final score of 1 over 83. Similarly to the OPU EES, the section that contained the not satisfied requirement in both trials was the “Harm–benefit evaluation of the procedure”. The specific item was related to possible adverse side effects that can lead to biomaterial damage, even if all precautionary measures were taken.

#### 3.1.2. ERA

The application of the OPU ERA first version, in a European zoo in October 2019, resulted in “low risk”. Checklists filled in by the three respondents were analyzed for assessing both the procedure itself and the effectiveness of communication among the participants. In particular, the assessment of the procedure itself did not find any relevant nonconformity in the procedures. All potential issues were taken into account and suitable measures were enforced to minimize risks. The only negative score was concerning “previous experience of the local team” in OPU on rhinos, which was not a problem in itself because of the presence of the B*io*R*escue* veterinary staff, who coordinated and carried out the procedures.

When the answers of all three respondents were analyzed to assess communication, the obtained risk score was 57, over a total of 190. The “low risk” ranking notwithstanding, the second and third review levels were applied, and the ERA outcome was further investigated. Twenty items—distributed among the A and D phases—were identified. The characteristics of the requirements not met were related to “Documents, procedures, operating instructions” for 10 items and “Operational requirements” for the other 10 items. Apart from “experience of the local team”, in all these cases, the problem was that the two local respondents did not answer to some items, although the B*io*R*escue* veterinarian had, so the modal value was 0. The same was true of the whole of phase E. Thanks to the third review level, it was possible to detect that the items that recorded “no” or “no answer” were mainly related to sub-optimal explicit communication of some issues between the three main people responsible for the procedure.

The highlighted communication issues in the first version were not detected in the second one. Consequently, the OPU ERA applied in December 2019 in Kenya resulted in “low risk” with a risk score of 0 over 220. Therefore, it was not necessary to proceed with the second and third review levels (Table 9).

The application of the IVF-lab ERA, in October 2019, resulted in “low risk”, with a risk score of 0 over 184 (Table 10). All the requirements’ characteristics related to “Factors affecting the process (documental and procedural support aspects), “Factors related to the traceability and distribution of specimens, laboratory operator’s safety, quality and availability of laboratory facilities” and “Factors related to the viability of gametes and embryos and to the instrumental requirements and the chemical reagents used” were met for the rhinoceroses’ biomaterial safety. It was not necessary to proceed with the second and third review levels. Therefore, there was no need to perform a second assessment trial after addressing problematic issues.

Of course, also having established the inclusion of an ethical self-assessment in ART procedures as a routine protocol is to be considered in itself as an improvement of the procedures, as it ensures the high standards of the procedures themselves.

### 3.2. How Applying the Tool in Actual Field Conditions Improved the Tool Itself

As already explained, the tool is designed to be able to incorporate changes allowing it to be refined by means of consultation between ethicists, scientists and stakeholders following each application of it. After the application of the first version of the tool to the OPU procedure, some areas needing further addressing in the ERA and EES checklists were highlighted. The items added as a consequence of the process in the OPU EES and OPU ERA are shown in Table 11. The items added to the OPU EES were also added to the IVF-lab EES as they were also relevant to the IVF procedure. 

## 4. Discussion

The application of the ETHAS to the procedures performed during the present study both contributed to the overall acceptability of the project and improved communication among the projects’ partners while refining the tool itself, in view of its standardization and application to other contexts in which ARTs are used for mammalian conservation projects.

Regarding the procedures assessed in the present study, it is important to note how having applied a tool which integrated risk assessment (general, ethical, welfare), pain/distress/welfare evaluation, harm–benefit analysis and the 3Rs tenet more likely had the potential to make the assessment and, eventually, help in the detection of problematic issues than using only one of these approaches separately. If we analyze, in more depth, the results of the ETHAS assessment, the harm–benefit analysis part allowed highlighting both positive effects and harms that could be generated by the execution of the OPU and IVF-lab procedures on wild animals and their specimens. Among positive effects highlighted during the assessment were: routine health and welfare check-up of the animals involved; the possibility of propagation of the genetic material of the specimens involved; scientific knowledge and know-how improvements that might find positive applications in other fields; the development of new technologies and procedures to promote the health and welfare of wild animals; the development of protocols for the conservation of endangered wild species. It was also possible to check whether the B*io*R*escue* team was committed to sharing the outcoming benefits with local communities. The restoration of the NWR’s wild populations can directly positively affect local communities’ economies through tourism and indirectly improve the quality of local communities’ lives, restoring the African ecosystem and landscape [113,114]. The ETHAS confirmed that the know-how deriving from the procedures’ optimization was shared with local veterinarians.

The local staff was also directly involved in the compilation of the OPU ERA, since a general and comprehensive goal of the ETHAS is to facilitate discussion among participants. The testing of the ETHAS confirmed that the tool was effective in this respect. As the results of the OPU ERA checklists showed, after the first application, the issues with negative answers caused by a lack of communication were not detected in the second one. In general, better communication among participants helps to avoid, reduce or manage the risks of the procedures and to guarantee high standards. The application of the ETHAS to the laboratory procedures contributed to guarantee high standards also in the IVF procedure and to safeguard the biomaterial involved, as the three embryos created by NWRs are of exceptionally high conservation value.

Through the ETHAS, it was also possible to check for potential harms that may occur during the procedures and if everything possible was done to avoid their occurrence. The main potential harms highlighted by ETHAS application mostly concern the possible side effects of the veterinary procedures on the animals’ health and welfare, correct preservation of the biomaterial and staff safety. However, since potential risks might occur during the veterinary procedures on wild animals, ETHAS application allowed highlighting the above-mentioned critical points, investigating whether action plans have been developed to deal with them and facilitating discussion around them between the staff members.

With regard to the animal welfare issues involved in the procedure, as highlighted by the positive results of the items specifically designed in the OPU ERA and EES, it was found that the team was committed to preserve and protect animal welfare, by monitoring the animals before, during and after the procedures, through physiological and behavioral analyses. Moreover, even if scientific evidence shows that the OPU procedure can be repeated on the same animal several times, the ETHAS allowed for checking if an adequate time-lapse between procedures was respected, as dictated by the best veterinary practices. Furthermore, specific items of the ERA checklist were included in order to analyze the welfare of other animals not directly involved in the procedures, such as herd mates sharing the same facilities or even enclosures.

Implementation of the 3Rs was another purpose of the ETHAS. Results showed that refinement, reduction and replacement were applied in the procedures whenever possible. For instance, refinement was applied by developing a new instrument for oocytes pick-up in rhinoceroses and by improving the procedures and techniques, with the aim of increasing the welfare of the animals involved, the efficacy of the procedures and the correct preservation of specimens. Another aspect related to refinement was the inclusion of items regarding environmentally friendly waste disposal in the EES, after the first trial. The replacement of laboratory media with synthetic ones, the replacement of materials with lower environmental impact and the replacement of procedures and equipment with a lower impact on animal welfare were considered and applied whenever possible. Finally, reduction was implemented by maximizing the number of sampling procedures under the same anesthesia to reduce the number of veterinary interventions as much as possible.

Furthermore, the applications of the ETHAS in different conditions (zoos and semi-captive management) have contributed to refine the accuracy and inclusiveness of the tool itself. OPU and IVF-lab ERAs underwent several applications that allowed improving the tool via a shared work between ethicists and experts. This process permitted reviewing and refining the checklists iteratively through a participative approach.

Last, but not least, a general and comprehensive goal of the ETHAS was to assist scientists to carry out a self-assessment in addressing ethical evaluation of ART application in conservation projects. The results of the present study show that the application of such an ongoing assessment was effective in ensuring the high standards of the procedures, including respect for animal welfare, and facilitating effective communication among participants. It is important to note that the application of a form of ethical self-assessment to procedures or projects constitutes in itself a contribution to their acceptability even if no problematic issue is detected. All this is a value in itself and can increase acceptance of this kind of project by the public.

Limitations and Future Developments

Self-assessment can also be seen as the main limit of ETHAS application, as the evaluation process can be interpreted as self-referential. Nevertheless, as already pointed out, the primary function of ethical self-assessment is to help scientists think, in detail and proactively, through ethical issues surrounding their research. Usually, ethical evaluation regarding conservation projects, when it is performed, is made by an external authority, which gives a general ethical approval to the overall project before it starts. On the contrary, ethical self-assessment offers the opportunity for an ongoing detailed scrutiny of all the main ethical aspects involved in the project, including the procedures that are carried out on animals, being proactive in detecting hazards for their welfare and taking measures to minimize them beforehand. In general, ethical self-assessment allows for a comprehensive and transparent evaluation process which can also be communicated to the public.

Another difficulty in applying such tool is the balancing between the need for standardization and that for customizing procedures and situations. Moreover, the fact that the tool is designed to evolve through iterative confrontation makes standardization more difficult. Notwithstanding, the ETHAS will continuously be tested in different contexts, species and procedures, in order to increase the comprehensiveness of the tool. However, it is important to note that the general frame and most of the tool are already adaptable to a more general use in different contexts, species and procedures, such as semen collection, embryo transfer, surrogate pregnancy and birth management, and to other innovative procedures regarding stem cell-associated techniques.

## 5. Conclusions

Ethical assessment of the application of ARTs in conservation is important for many reasons. In conservation breeding programs, for instance, animal welfare is a crucial element to be considered, alongside safety for the people involved and the quality of the procedures. Moreover, ethical assessment—especially when performed in the guise of self-assessment—allows anticipating the critical aspects that can compromise the ethical acceptability of a procedure and intervening before their eventual occurrence could damage the reputation of the whole conservation project and alienate societal support. As ARTs will become ever more important for conservation, the need to expand and deepen the ethical research on this topic will increase. An exemplary case, in this sense, is provided by the B*io*R*escue* project, which, alongside the development and testing of new approaches in the conservation of a “technically extinct” species, implemented a self-assessment tool designed for improving the procedures from an ethical standpoint. The application of such a tool within the project allowed for the mutual goals of improving some aspects of the communication among the projects’ partners and improving the tool itself, to be applied in the near future to other contexts in which ARTs are applied for the conservation of other mammal species. Despite the obvious advantages of this kind of self-assessment, such an approach is almost underestimated in the literature dealing with ART in conservation, as shown by a simple Scopus search on the subject. Therefore, tools such as the ETHAS could raise the ethical standards of applications of ARTs to conservation and, in this way, contribute to their success.

## Figures and Tables

**Table 1:** Ethical Evaluation Sheet sections and bibliography.

EES Sections and Sub-Sections		Number of Items (Sub-Items) OPU EES		Number of Items (Sub-Items) IVF-Lab EES		Bibliography
		1st Trial	2nd Trial	1st Trial	2nd Trial	
(A)	Documents					[21,39,43,67,68,69,70,71,72,73]
		11 (13)	11 (13)	9 (10)	9 (10)	
(B)	Harm–benefit evaluation of the procedure					[36,64,65,66,69,74,75,76,77,78,79,80,81]
(B1)	Benefit evaluation	12 (14)	12 (14)	7 (7)	7 (7)	
(B2)	Harm evaluation	8 (9)	8 (9)	4 (8)	4 (8)	
(C)	Procedure Quality Evaluation					[21,36,39,40,54,64,65,66,75,76,77,78,79,80,81,82]
(C1)	Pre-screening consideration	6 (6)	6 (6)	6 (6)	6 (6)	
(C2)	Procedural steps evaluation	3 (3)	3 (3)	5 (5)	5 (5)	
(C3)	3Rs evaluation (replacement, reduction, refinement)	23 (23)	23 (23)	14 (21)	14 (21)	
(D)	Scientific team quality evaluation					[62,64,76]
(D1)	Team and teamwork	13 (13)	14 (14)	12 (17)	12 (17)	
(D2)	Equipment	5 (5)	7 (7)	4 (4)	6 (6)	
(D3)	Laboratories and biobanks	2 (2)	2 (2)	3 (3)	3 (3)	
(E)	Final ethical evaluation of the procedure					[76]
		11 (11)	11 (11)	9 (9)	9 (9)	

**Table 2:** Acceptability ranking and scoring of the ovum pick-up (OPU) and in vitro fertilization (IVF-lab) Ethical Evaluation Sheets (EESs) applied in the present study.

Acceptability Ranking	Score in OPU EES		Score in IVF-Lab EES	
	1st Version	2nd Version	1st Version	2nd Version
Totally acceptable	0–29	0–30	0–27	0–27
Partially acceptable	30–58	31–60	28–54	28–55
Not acceptable	59–88	61–91	55–81	56–83

**Table 3:** OPU Ethical Risk Assessment (ERA) phases and bibliography.

OPU Ethical Risk Assessment Phases		Number of Items (Sub-Items) 1° Version	Number of Items (Sub-Items) 2° Version	Bibliography
(A)	Identification of the individual/s, welfare assessment and procedure planning	17 (34)	19 (36)	[19,67,84,85,86,87,88]
(B)	Ovarian stimulation protocol	6 (8)	6 (8)	[50,54,89]
(C)	Anesthetic procedure for oocyte recovery	10 (18)	13 (27)	[50,52,53,54,55,56,57,89,90,91]
(D)	Oocyte recovery by transrectal procedure	12 (20)	11 (19)	[50,54,90,92,93,94]
(E)	Gametes packaging	7 (11)	7 (11)	[95,96,97]

**Table 4:** IVF-lab ERA phases and bibliography.

IVF-Lab ERA Phases		Number of Items (Sub-Items)	Bibliography
(A)	Laboratory quality assessment and specimens processing	17 (32)	[98,99,100,101]
(B)	Gametes shipping to laboratory	7 (8)	[7,54,102,103,104]
(C)	Gametes biobanking	7 (8)	[7,102,105,106]
(D)	Gametes preparation for ICSI	13 (16)	[54,55,56,57,58,59,60,61,62,63,64,65,66,67,68,69,70,71,72,73,74,75,76,77,78,79,80,81,82,83,84,85,86,87,88,89,90,91,92,93,94,95,96,97,98,99,100,101,102,103,104,105,106,107]
(E)	Intracytoplasmic sperm injection (ICSI)	6 (6)	[54,92,107,108]
(F)	Embryos culture	7 (7)	[54,109]
(G)	Embryos cryopreservation and biobanking	4 (11)	[54,102,110]
(H)	Embryos packaging	4 (7)	[109]
(I)	Embryos shipping	7 (8)	[109,111]

**Table 5:** Description of risk categories and corresponding score used for phases A, B, C and D the OPU ERA and for phase E of the OPU ERA and all phases (A–I) of the IVF-lab ERA.

Phases	Categories	Characteristics of the Requirement	Score
OPU ERA (phases A–D)	Low	Documents, procedures, operating instructions, etc.	1
	Medium	Structural, instrumental and environmental requirements.	2
	High	Operational requirements.	3
OPU ERA (phase E) and IVF-lab ERA	Low	Factors affecting the process (documental and procedural support aspects).	1
	Medium	Factors related to the traceability and distribution of specimens, laboratory operator’s safety, quality and availability of laboratory facilities.	2
	High	Factors related to the viability of gametes and embryos and to the instrumental requirements and the chemical reagents used.	3

**Table 6:** Risk ranks of the OPU and IVF-lab ERAs.

Risk Rank	Score in OPU		Score in IVF-Lab
	1st Version	2nd Version	Final Version
	(October 2019)	(December 2019)	(October 2019)
Low	0–63	0–73	0–61
Medium	64–126	74–146	62–123
High	127–190	147–220	124–184

**Table 7:** Ethical Assessment Tool (ETHAS) overall final evaluation, obtained by combining results from the ESS and ERA checklists.

ERA ESS	Low Risk	Medium Risk	High Risk
Totally acceptable	Acceptable	Acceptable with mitigation	Not acceptable
Partially acceptable	Acceptable with mitigation	Acceptable with mitigation	Not acceptable
Not acceptable	Not acceptable	Not acceptable	Not acceptable

**Table 8:** EES results. Please note that the changes detailed in Section 3.2 were already included in the EES version used for the second OPU and IVF trials.

EES		OPU EES 1st Trial		OPU EES 2nd Trial		IVF-Lab EES 1st Trial		IVF-Lab EES 2nd Trial	
		Positive Answers	Negative Answers	Positive Answers	Negative Answers	Positive Answers	Negative Answers	Positive Answers	Negative Answers
(A)	Documents	13 over 13	0 over 13	13 over 13	0 over 13	10 over 10	0 over 10	10 over 10	0 over 10
(B)	Harm–benefit evaluation of the procedure	20 over 23	3 over 23	20 over 23	3 over 23	14 over 15	1 over 15	14 over 15	1 over 15
(C)	Procedure quality Evaluation	32 over 32	0 over 32	32 over 32	0 over 32	32 over 32	0 over 32	32 over 32	0 over 32
(D)	Scientific team quality evaluation	20 over 20	0 over 20	23 over 23	0 over 23	24 over 24	0 over 24	26 over 26	0 over 26
Total		85 over 88	3 over 88	88 over 91	3 over 91	80 over 81	1 over 81	82 over 83	1 over 83

**Table 9:** Results of the first and second assessment trials using the OPU ERA checklists. Please note that the changes detailed in Section 3.2. were already included in the ERA version used for the second OPU trial and that the results shown for OPU refer to the analysis of the answers of all three respondents.

OPU ERA Phases		1st Trial (October 2019)		2nd Trial (December 2019)	
		Positive Answers	Negative Score	Positive Answers	Negative Score
(A)	Animal selection, procedure planning and welfare	27 over 34	10 over 75	36 over 36	0 over 79
(B)	Ovarian stimulation protocol	8 over 8	0 over 21	8 over 8	0 over 21
(C)	Anesthetic procedure	15 over 18	7 over 37	27 over 27	0 over 66
(D)	Oocyte recovery by transrectal procedure	9 over 20	23 over 40	19 over 19	0 over 37
(E)	Gametes packaging	0 over 11	17 over 17	11 over 11	0 over 17
Total		59 over 91	57 over 190	101 over 101	0 over 220

**Table 10:** Description of IVF-lab ERA standard checklist application and results.

IVF-Lab ERA Phases		October 2019	
		Positive Answers	Negative Score
(A)	Laboratory quality assessment and specimens processing	32 over 32	0 over 54
(B)	Gametes shipping to laboratory	8 over 8	0 over 14
(C)	Gametes biobanking	8 over 8	0 over 17
(D)	Gametes preparation for ICSI	16 over 16	0 over 28
(E)	ICSI	6 over 6	0 over 16
(F)	Embryos culture	7 over 7	0 over 11
(G)	Embryos cryopreservation and Biobanking	11 over 11	0 over 19
(H)	Embryos packaging	7 over 7	0 over 11
(I)	Embryos shipping	8 over 8	0 over 14
Total		103 over 103	0 over 184

**Table 11:** Items added to the first OPU EES, OPU ERA and IVF-lab EES standard versions to obtain the second ones.

**New Added Items to OPU EES**
Have the aspects related to the environmental impact of the staff travels been considered and have measures been taken to decrease it? (i.e., use train instead of airplane whenever possible, contributing to a certified carbon offset program for flights)
Have the aspects related to the environmental impact of the equipment and materials been considered and have measures to decrease it been taken?
Have the aspects related to the waste deriving from the procedure been considered and have measures to decrease it been taken?
**New Added Items to OPU ERA**
If the animal or animals have already undergone the OPU procedure, were the procedure and the recovery of the animal carried out without difficulties?
Does the facility have an ethical internal committee?
Have measures/actions to avoid or minimise possible animal’s injuries due to its partial control of the awareness during (a) and (b) been planned? a) pre-anaesthesia b) post-anaesthesia recovery
Have measures/actions to avoid or minimise any animal distress or suffering, during a) and b), been planned? (a) pre-anaesthesia (b) post-anaesthesia
Are measures/actions to avoid or minimise the potential negative influence of (a), (b) and (c) on the welfare of the animal/s involved in the procedure been planned? (a) Visual/olfactory/auditory inputs from other individuals (b) Visual/olfactory/auditory absence of inputs from individual/s of the same social group (c) Absence of familiar keeper/s.
Are measures/actions to avoid or minimise the potential negative influence of (a), (b) and (c) on the welfare of other animal/s not directly involved in the procedure been planned? (a) Visual/olfactory/auditory inputs from other individuals (b) Visual/olfactory/auditory absence of inputs from individual/s of the same social group (c) Absence of familiar keeper/s.
**New Added Items to IVF-Lab EES**
Have the aspects related to the environmental impact of the equipment and materials been considered and have measures to decrease it been taken?
Have the aspects related to the waste deriving from the procedure been considered and have measures to decrease it been taken?

## Data Availability

Not applicable.

## Supplementary Materials

The following are available online at https://www.mdpi.com/2076-2615/11/2/312/s1, File S1: OPU EES_1st trial, File S2: OPU ERA_october2019, File S3: IVF-lab EES_1st trial, File S4: IVF-lab ERA.
