# Supplementary material for: An Ethical Assessment Tool (ETHAS) to Evaluate the Application of Assisted Reproductive Technologies in Mammals’ Conservation: The Case of the Northern White Rhinoceros (Ceratotherium simum cottoni)
Source: Animals (Basel). 2021 Jan 26;11(2):312. doi: 10.3390/ani11020312 (PMC7911958; doi:10.3390/ani11020312)
Supplement: Supplementary file 1 [file animals-11-00312-s001.zip › File S1.pdf]

OPU

ETHICAL EVALUATION SHEET

BioRescue Ethical Team

Ethics Laboratory for Conservation,
Veterinary Medicine and Animal Welfare

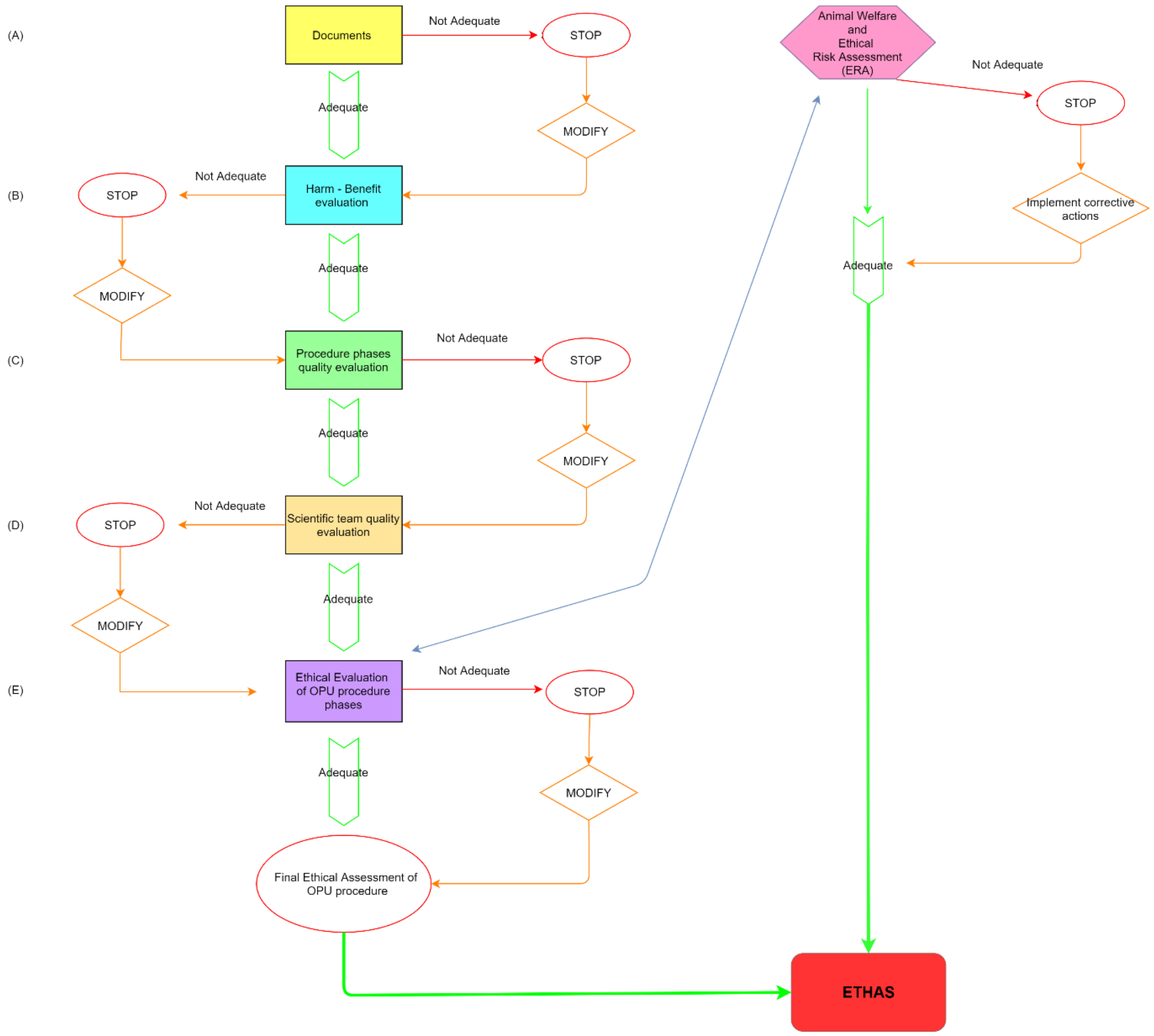

Figure 1: Flow chart of phases from A to E.

A	DOCUMENTS			
				Details when needed
1.	Has this OPU protocol been planned in compliance with the Directive 2010/63/EU?	YES	NO	…………………………
2.	Do the following govermentes support this OPU procedure?			…………………………
	a) EU governement	YES	NO	
	b) Non-EU goverments	YES	NO	
3.	Has the government of the country that supervises the project given economic support?	YES	NO	…………………………
4.	If animals, or biomaterials of low and/or lower-middle-income countries are involved, have documents showing compliance with the following normative been submitted to the competent authority:			…………………………
	a) Nagoya Protocol on access and benefit-sharing?	YES	NO	
	b) EU Regulation No 511/2014 (on compliance of Nagoya Protocol)?	YES	NO	
	Horizon 2020: "Resources from a non-EU country — Any use of local resources (especially animal and/or human tissue samples, genetic material, live animals, human remains, materials of historical value, endangered fauna or flora samples, fossils) must show respect for cultural traditions and share benefits (i.e. also benefit local participants and their communities, involve local workers – as equal partners – and respond to local worker's needs)."			
5.	If the OPU procedure is part of a project which involves low and/or lower-middle-income countries, are benefit-sharing measures been planned?	YES	NO	…………………………

Ethics Laboratory for Veterinary Medicine, Conservation, and Animal Welfare, 2019. All rights reserved. Any use, in whole or in part, of the contents included in this document, including the storage, reproduction, reprocessing, dissemination or distribution of the contents through any technological platform, support or electronic network, without the prior written authorization of all the authors is prohibited.

	Please, give details on:<br>a) Benefit sharing measures.<br>b) Responsiveness to local population needs.<br><br>a) ……………………………………………………<br>……………………………………………………<br>……………………………………………………<br>b) ……………………………………………………<br>……………………………………………………<br>……………………………………………………			
6.	If needed, have export licenses, to import biomaterial from non-EU countries into the EU, been obtained?<br><br>*Horizon 2020 "Import/export of material — If genetic resources are transferred across borders, it may be mandatory under the law of the provider country to obtain an authorisation for the transfer. In addition, you must use an agreement which describes the conditions for the export and the terms of utilisation and, if applicable, relevant benefit-sharing measures."*	YES	NO	……………………………<br>……………………………<br>……………………………<br>……………………………<br>……………………………
7.	Has CITES authorization from exporting and importing countries for the supply of endangered animal species been obtained?	YES	NO	……………………………<br>……………………………<br>……………………………<br>……………………………<br>……………………………
8.	Has European Endangered Species Programme (EEP) authorization for the use of endangered animal species been obtained?	YES	NO	……………………………<br>……………………………<br>……………………………<br>……………………………<br>……………………………
9.	Has an ethical approval been obtained?	YES	NO	……………………………<br>……………………………<br>……………………………<br>……………………………<br>……………………………
10.	Have the licenses to practice the profession of veterinary medicine of all the veterinarians involved in the procedure been submitted?	YES	NO	……………………………<br>……………………………<br>……………………………<br>……………………………<br>……………………………
11.	Have certificates of good standing been submitted?<br><br>*(e.g. a certification that no measures of disciplinary action of professional or administrative nature had been taken for all the staff involved in the procedure)*	YES	NO	……………………………<br>……………………………<br>……………………………<br>……………………………<br>……………………………

Ethics Laboratory for Veterinary Medicine, Conservation, and Animal Welfare, 2019. All rights reserved. Any use, in whole or in part, of the contents included in this document, including the storage, reproduction, reprocessing, dissemination or distribution of the contents through any technological platform, support or electronic network, without the prior written authorization of all the authors is prohibited.

B	HARM - BENEFIT EVALUATION			
**B1**	**Benefit evaluation**			
				Details when needed
12.	Is the OPU procedure part of a novel conservation strategy?	YES	NO	……………………… ……..……………… ……………………… …………..……….. ………………………
13.	Will the application of the OPU procedure improve knowledge in developing new conservation strategies?	YES	NO	……………………… ……..……………… ……………………… …………..……….. ………………………
14.	Could the effects of the OPU procedure, as part of a novel conservation strategy, have a positive impact on the natural habitat in the future?	YES	NO	……………………… ……..……………… ……………………… …………..……….. ………………………
15.	Will the scientific improvements of the OPU procedure impact positively on other scientific fields?	YES	NO	……………………… ……..……………… ……………………… …………..……….. ………………………
16.	Will the application of the OPU procedure, as part of a conservation project, produce an economic benefit in the future for local populations?	YES	NO	……………………… ……..……………… ……………………… …………..……….. ………………………
17.	Will the application of the OPU procedure improve the professional training of laboratory technicians?<br>If yes, please give details<br>……………………………………………………………… ……………………………………………………………… ………………………………………………………………	YES	NO	……………………… ……..……………… ……………………… …………..……….. ………………………
18.	Will the application of the OPU procedure have a capacity building component?	YES	NO	……………………… ……..……………… ……………………… …………..……….. ………………………

Ethics Laboratory for Veterinary Medicine, Conservation, and Animal Welfare, 2019. All rights reserved. Any use, in whole or in part, of the contents included in this document, including the storage, reproduction, reprocessing, dissemination or distribution of the contents through any technological platform, support or electronic network, without the prior written authorization of all the authors is prohibited.

19.	Literature says infertility is a widespread phenomenon in managed wildlife. Is it that true for the following populations:			………………………… ………..……………… ………………………… ……………..………. …………………………
	a) Captive population of southern white rhinos?	YES	NO	
	b) Wild population of southern white rhinos?	YES	NO	
	c) Captive population of northern white rhinos?	YES	NO	
20.	Will the health of the animals of this or other related species benefit from the scientific/technological improvements that might be obtained?	YES	NO	………………………… ………..……………… ………………………… ……………..………. …………………………
21.	Could the OPU procedure or part of it have positive side effects on the animal under the procedure?<br>If yes, which ones?<br>……………………………………………………………… ……………………………………………………………… ………………………………………………………………	YES	NO	………………………… ………..……………… ………………………… ……………..………. …………………………
22.	Could the OPU procedure or part of it have positive side effects on the species?<br>If yes, which ones?<br>……………………………………………………………… ……………………………………………………………… ………………………………………………………………	YES	NO	………………………… ………..……………… ………………………… ……………..………. …………………………
23.	Is the scientific knowledge achievable from the OPU procedure optimization/application relevant to the assisted reproductive technology (ART) for mammals?	YES	NO	………………………… ………..……………… ………………………… ……………..………. …………………………

Ethics Laboratory for Veterinary Medicine, Conservation, and Animal Welfare, 2019. All rights reserved. Any use, in whole or in part, of the contents included in this document, including the storage, reproduction, reprocessing, dissemination or distribution of the contents through any technological platform, support or electronic network, without the prior written authorization of all the authors is prohibited.

B2	Harm evaluation			
				Details when needed
24.	Could the OPU procedure, or part of it, have any adverse side effects on the animal/s under procedure?<br><br>If yes, which ones?<br>……………………………………………………………<br>……………………………………………………………<br>……………………………………………………………	YES	NO	………………………<br>………..……………<br>………………………<br>…………….………<br>………………………
25.	Could the OPU procedure, or part of it, have any adverse side effects on the species?<br><br>If yes, which ones?<br>……………………………………………………………<br>……………………………………………………………<br>……………………………………………………………	YES	NO	………………………<br>………..……………<br>………………………<br>…………….………<br>………………………
26.	Has a plan for the mitigation of the adverse effects that the animals might experience, during the following a) and b) steps, been set up?			………………………<br>………..……………<br>………………………<br>…………….………<br>………………………
	a) Anesthetic protocol (safe immobilization or sedation, anesthesia, recovery)	YES	NO	
	b) OPU	YES	NO	
	If yes, give details<br>a) ………………………………………………………<br>………………………………………………………<br>………………………………………………………<br>b) ………………………………………………………<br>………………………………………………………<br>………………………………………………………			
27.	Have possible adverse effects of the specimens transport been adequately considered?	YES	NO	………………………<br>………..……………<br>………………………<br>…………….………<br>………………………

Ethics Laboratory for Veterinary Medicine, Conservation, and Animal Welfare, 2019. All rights reserved. Any use, in whole or in part, of the contents included in this document, including the storage, reproduction, reprocessing, dissemination or distribution of the contents through any technological platform, support or electronic network, without the prior written authorization of all the authors is prohibited.

28.	In case of adverse effects during the oocytes transport, has a mitigation plan been set up?	YES	NO	………………………… ………..……………… ………………………… ……………..……….. …………………………
29.	Has a plan for the mitigation of the adverse effects that the animal might experience after the OPU procedure phases been prepared?<br><br>If yes, give details<br><br>……………………………………………………………… ……………………………………………………………… ………………………………………………………………	YES	NO	………………………… ………..……………… ………………………… ……………..……….. …………………………
30.	In case of critical situations during the OPU procedure, have the possible negative effects (public or scientific opinions, etc.) been considered and discussed within the project partners?	YES	NO	………………………… ………..……………… ………………………… ……………..……….. …………………………
31.	Is there any other concern about the harm evaluation of the procedure you would like to underline?<br><br>If yes, give details<br>……………………………………………………………… ……………………………………………………………… ………………………………………………………………	YES	NO	………………………… ………..……………… ………………………… ……………..……….. …………………………

Ethics Laboratory for Veterinary Medicine, Conservation, and Animal Welfare, 2019. All rights reserved. Any use, in whole or in part, of the contents included in this document, including the storage, reproduction, reprocessing, dissemination or distribution of the contents through any technological platform, support or electronic network, without the prior written authorization of all the authors is prohibited.

C	PROCEDURE QUALITY EVALUATION			
**C1**	**Pre-screening considerations**			
				Details when needed
32.	Has the equipment for the OPU procedure phases been already tested on animals in other related studies?<br><br>If yes, give details<br>……………………………………………………………<br>……………………………………………………………<br>……………………………………………………………	YES	NO	…………………………<br>…………………………<br>…………………………<br>…………………………<br>…………………………
33.	Since OPU procedure is commonly used in farm and domestic animals, has the necessary species-specific adjustments being considered?	YES	NO	…………………………<br>…………………………<br>…………………………<br>…………………………<br>…………………………
34.	Has the opportunity to modify any step of the standard ART protocol - used in farm and domestic animals - ever been considered to improve the procedure?<br><br>If yes, give details<br>……………………………………………………………<br>……………………………………………………………<br>……………………………………………………………	YES	NO	…………………………<br>…………………………<br>…………………………<br>…………………………<br>…………………………
35.	Has a procedural timetable for each single phase of the procedure been drawn up?	YES	NO	…………………………<br>…………………………<br>…………………………<br>…………………………<br>…………………………
36.	Are project partners committed to eventually publishing also critical events so that they could be avoided in future projects?<br><br>If no, explain why<br>……………………………………………………………<br>……………………………………………………………<br>……………………………………………………………	YES	NO	…………………………<br>…………………………<br>…………………………<br>…………………………<br>…………………………
37.	Has it been planned to share and publish relevant data about the application of procedures refinement?<br><br>If no, explain why.<br>………………………………………………………<br>………………………………………………………<br>………………………………………………………	YES	NO	…………………………<br>…………………………<br>…………………………<br>…………………………<br>…………………………

Ethics Laboratory for Veterinary Medicine, Conservation, and Animal Welfare, 2019. All rights reserved. Any use, in whole or in part, of the contents included in this document, including the storage, reproduction, reprocessing, dissemination or distribution of the contents through any technological platform, support or electronic network, without the prior written authorization of all the authors is prohibited.

C2	Procedural steps evaluation			
				Details when needed
38.	Has the anaesthetic protocol been tested and evaluated in any other related species?<br><br>If yes, give details on the possible side effects:<br><br>……………………………………………………………… ……………………………………………………………… ………………………………………………………………	YES	NO	………………………… ……..……………………… ………………………… ……..……………………… …………………………
39.	Has the OPU procedure been tested and evaluated in any other related species?<br><br>If yes, give details on the possible side effects:<br>……………………………………………………………… ……………………………………………………………… ………………………………………………………………	YES	NO	………………………… ……..……………………… ………………………… ……..……………………… …………………………
40.	Since the OPU procedure has been tested and evaluated in other related species, has a variation on the ovum collection efficiency between the northern white rhinoceroses and the southern white rhinoceroses been considered?<br><br>……………………………………………………………… ……………………………………………………………… ………………………………………………………………	YES	NO	………………………… ……..……………………… ………………………… ……..……………………… …………………………

C3	3Rs evaluation			
Replacement				
				Details when needed
41.	Do the animals undergo to a physiological and behavioural assessment after performing the OPU procedure?<br><br>If yes, for how long and who will be in charge of it?<br>………………………………………………………… ………………………………………………………… …………………………………………………………	YES	NO	………………………… ……..……………………… ………………………… ……..……………………… …………………………

Ethics Laboratory for Veterinary Medicine, Conservation, and Animal Welfare, 2019. All rights reserved. Any use, in whole or in part, of the contents included in this document, including the storage, reproduction, reprocessing, dissemination or distribution of the contents through any technological platform, support or electronic network, without the prior written authorization of all the authors is prohibited.

42.	Have alternative procedural steps or parts of them been considered for the execution of the procedure?<br>If not, why?<br>If yes, explain.<br>…………………………………………………………<br>…………………………………………………………<br>…………………………………………………………	YES	NO	……………………………<br>……………………………<br>……………………………<br>……………………………<br>……………………………
43.	Has the opportunity to change some procedural steps for an incomplete replacement been considered?<br>If yes, give some details.<br>…………………………………………………………<br>…………………………………………………………<br>…………………………………………………………<br>*(for examples, has it been considered to include steps to amplify the results of the procedure, so that it will be not necessary to repeat it several times. i.e. using more efficient hormone stimulation protocols, steps of cell reproduction in vitro, etc.).*	YES	NO	……………………………<br>……………………………<br>……………………………<br>……………………………<br>……………………………
Reduction				
44.	Has this procedure been optimized and standardized so to reduce to a minimum the number of animals involved?	YES	NO	……………………………<br>……………………………<br>……………………………<br>……………………………<br>……………………………
45.	Has the possibility of reducing animal manipulation time during the procedure been considered?<br>If not, why?<br>…………………………………………………………<br>…………………………………………………………<br>…………………………………………………………	YES	NO	……………………………<br>……………………………<br>……………………………<br>……………………………<br>……………………………
46.	Will the specimens of the animals involved in this project be shared with other scientific groups?<br>If yes, give details.<br>If no, give details.<br>…………………………………………………………<br>…………………………………………………………<br>…………………………………………………………	YES	NO	……………………………<br>……………………………<br>……………………………<br>……………………………<br>……………………………

Ethics Laboratory for Veterinary Medicine, Conservation, and Animal Welfare, 2019. All rights reserved. Any use, in whole or in part, of the contents included in this document, including the storage, reproduction, reprocessing, dissemination or distribution of the contents through any technological platform, support or electronic network, without the prior written authorization of all the authors is prohibited.

47.	Is it necessary to repeat the OPU procedure several times on the same animal or on other related species or subspecies?<br><br>If yes, please, explain why<br><br>……………………………………………………………<br>……………………………………………………………<br>……………………………………………………………	YES	NO	…………………………<br>…………………………<br>…………………………<br>…………………………<br>…………………………
48.	If the animals that undergo the OPU procedure have been already involved in previous procedures, have the suffering or the distress that the animals could have been experienced during the previous procedures, been evaluated?<br><br>*(Art. 16 Directive 2010/63/EU) An animal already used in one or more procedures " may only be reused in a new procedure provided that the following conditions are met: (a) the actual severity of the previous procedures was 'mild' or 'moderate'; (b) it is demonstrated that the animal's general state of health and well-being has been fully restored".*	YES	NO	…………………………<br>…………………………<br>…………………………<br>…………………………<br>…………………………
49.	Will it be collected, from the animal under the OPU procedure, as many as possible samples (ova, tissues, *etc*.) that might be of interest in this project or others in the future?	YES	NO	…………………………<br>…………………………<br>…………………………<br>…………………………<br>…………………………
Refinement				
50.	Has the OPU procedure ever been used in other wild mammal species, so that most of the risks have already been taken into account, analysed and minimized?	YES	NO	…………………………<br>…………………………<br>…………………………<br>…………………………<br>…………………………
51.	Have alternative procedural steps or part of them that might reduce pain, suffering, or distress of the animal involved ever been considered?	YES	NO	…………………………<br>…………………………<br>…………………………<br>…………………………<br>…………………………
52.	Have methods/procedures to reduce or avoid pain, suffering, distress, or lasting harm to animals been planned?	YES	NO	…………………………<br>…………………………<br>…………………………<br>…………………………<br>…………………………

Ethics Laboratory for Veterinary Medicine, Conservation, and Animal Welfare, 2019. All rights reserved. Any use, in whole or in part, of the contents included in this document, including the storage, reproduction, reprocessing, dissemination or distribution of the contents through any technological platform, support or electronic network, without the prior written authorization of all the authors is prohibited.

53.	Have the innovative devices developed for the procedure been patented?	YES	NO	....
54.	Has the need to stop the OPU procedure prior to the planned completion schedule been considered?	YES	NO	....
55.	If an unexpected event occurs when the procedure can not be stopped, have alternative strategies been set up?	YES	NO	....
56.	In emergency case, has a protocol for euthanasia been adequately planned?	YES	NO	....
57.	In an emergency case, is someone in charge of euthanasia been identified?	YES	NO	....
58.	Has animal training for veterinary manipulation (blood collection, injections, etc.) been planned?	YES	NO	....
59.	Does the management of the animals remain unchanged during the procedure period?<br>If yes, give details.<br>..<br>..<br>..	YES	NO	....
60.	Has a person been designed to monitor the welfare and care of the animals during the procedure?	YES	NO	....

Ethics Laboratory for Veterinary Medicine, Conservation, and Animal Welfare, 2019. All rights reserved. Any use, in whole or in part, of the contents included in this document, including the storage, reproduction, reprocessing, dissemination or distribution of the contents through any technological platform, support or electronic network, without the prior written authorization of all the authors is prohibited.

61.	Will the animal wellbeing be monitored and assessed throughout all the OPU procedure period (before, during and after the procedure)?<br>If yes, explain how this will be done.<br>………………………………………………………………<br>………………………………………………………………	YES	NO	……………………………<br>……………………………<br>……………………………<br>……………………………<br>……………………………
62.	To avoid animal stress during the procedure, have the external variables (open field conditions, temperature, humidity, ventilation, lighting, and sound) been considered?	YES	NO	……………………………<br>……………………………<br>……………………………<br>……………………………<br>……………………………
63.	Are all the animals involved familiar with the area where the procedure will be carried out?	YES	NO	……………………………<br>……………………………<br>……………………………<br>……………………………
**D**	**SCIENTIFIC TEAM QUALITY EVALUATION**			
**D1**	**Team and Teamwork**			
				Details when needed
64.	In case of necessity, are the team's economic resources adequate for the procedure completation and to overcome problematic situations or emergencies?	YES	NO	……………………………<br>……………………………<br>……………………………<br>……………………………
65.	Are all the members of the veterinary team and all the other staff members involved in the OPU procedure experienced in this kind of procedures?	YES	NO	……………………………<br>……………………………<br>……………………………<br>……………………………<br>……………………………
66.	Are all the members of the veterinary team and all the other staff members involved in the OPU procedure continuously trained for this kind of procedure?	YES	NO	……………………………<br>……………………………<br>……………………………<br>……………………………<br>……………………………
67.	Are all the members of the veterinary team and all the other staff members involved in the OPU procedure trained to work with this kind of animals?	YES	NO	……………………………<br>……………………………<br>……………………………<br>……………………………<br>……………………………
68.	Has the team already taken part in relevant and innovative reproductive procedures?	YES	NO	……………………………<br>……………………………<br>……………………………<br>……………………………<br>……………………………

Ethics Laboratory for Veterinary Medicine, Conservation, and Animal Welfare, 2019. All rights reserved. Any use, in whole or in part, of the contents included in this document, including the storage, reproduction, reprocessing, dissemination or distribution of the contents through any technological platform, support or electronic network, without the prior written authorization of all the authors is prohibited.

69.	Has the team already developed innovative methodologies?	YES	NO	…………………………<br>…………………………<br>…………………………<br>…………………………<br>…………………………
70.	Is someone deciding the team members of the OPU procedure?<br>If yes, give details.<br>……………………………………………………<br>……………………………………………………<br>……………………………………………………	YES	NO	…………………………<br>…………………………<br>…………………………<br>…………………………<br>…………………………
71.	Do all the staff members have defined roles and clear responsibilities in the OPU procedure phases?	YES	NO	…………………………<br>…………………………<br>…………………………<br>…………………………<br>…………………………
72.	Have actions to mitigate the possible risks for the staff involved during the OPU procedure been considered?<br>If yes, which one?<br>……………………………………………………<br>……………………………………………………<br>……………………………………………………	YES	NO	…………………………<br>…………………………<br>…………………………<br>…………………………<br>…………………………
73.	Is the staff able to recognize any sign of psycho/physical discomfort of the animals involved in the procedure and trained to deal with it?	YES	NO	…………………………<br>…………………………<br>…………………………<br>…………………………<br>…………………………
74.	Is someone in charge of the transport of ova from where OPU procedure takes place to the assigned BioBank?<br>If yes, how has he/she been identified?<br>……………………………………………………<br>……………………………………………………<br>……………………………………………………	YES	NO	…………………………<br>…………………………<br>…………………………<br>…………………………<br>…………………………
75.	Is there a project manager responsible for the OPU procedure, who supervises and coordinates all the procedural phases?	YES	NO	…………………………<br>…………………………<br>…………………………<br>…………………………<br>…………………………

Ethics Laboratory for Veterinary Medicine, Conservation, and Animal Welfare, 2019. All rights reserved. Any use, in whole or in part, of the contents included in this document, including the storage, reproduction, reprocessing, dissemination or distribution of the contents through any technological platform, support or electronic network, without the prior written authorization of all the authors is prohibited.

76.	Has a person responsible for the stocking of NWR biological material in the BioBank been assigned?	YES	NO	....
**D2**	**Equipment**			
				Details when needed
77.	Is the adequate equipment, necessary for the proper conduction of all the OPU procedure phases, available? (including instruments for general and specific physical examination and diagnostic kits for specific procedure)	YES	NO	....
78.	Is the equipment adequate for the species under the OPU procedure?	YES	NO	....
79.	Has the equipment for correct conduction of the OPU procedure and administration of the treatments been evaluated safe for animal use?	YES	NO	....
80.	Before starting the OPU procedure, is the availability of personal protective equipment (PPE) for all the staff members involved checked?	YES	NO	....
81.	Has a control checklist been developed to verify if all the equipment needed for the procedure are available, correctly operating and sterilized?	YES	NO	....
**D3**	**Laboratories and BioBanks**			
				Details when needed
82.	Has a standardized protocol for the specimens processing been developed for the BioBank?	YES	NO	....
83.	If possible, are the specimens divided into aliquots and stored in more than one laboratory?	YES	NO	....

Ethics Laboratory for Veterinary Medicine, Conservation, and Animal Welfare, 2019. All rights reserved. Any use, in whole or in part, of the contents included in this document, including the storage, reproduction, reprocessing, dissemination or distribution of the contents through any technological platform, support or electronic network, without the prior written authorization of all the authors is prohibited.

E	ETHICAL EVALUATION OF THE PROCEDURE			
				Details when needed
84.	Has an OPU Ethical Risk Assessment been planned for each application of the procedure?	YES	NO	………………………… ………………………… ………………………… ………………………… …………………………
85.	For each OPU procedure's application, will the checklist be filled by:			………………………… ………………………… ………………………… ………………………… …………………………
	IZW team's member	YES	NO	
	Local veterinarian	YES	NO	
	Managing Director	YES	NO	
	Other	YES	NO	
86.	In case of disagreement regarding answers to a specific item, is it planned to discuss the options in order to find an agreement among the team members?	YES	NO	………………………… ………………………… ………………………… ………………………… …………………………
87.	In the case of a non-satisfied requirement that could be underlined by the Ethical Risk Assessment, will risk mitigation actions be implemented?	YES	NO	………………………… ………………………… ………………………… ………………………… …………………………
88.	Does the procedure accomplish with all the International and National Regulations?	YES	NO	………………………… ………………………… ………………………… ………………………… …………………………
89.	Has the staff safety been adequately evaluated with dedicated items of the OPU Ethical Risk Assessment?	YES	NO	………………………… ………………………… ………………………… ………………………… …………………………

90.	Benefits deriving from the procedure application are:	Low	Medium	High
91.	The possibility of the procedure's successful accomplishment is:	Low	Medium	High

Ethics Laboratory for Veterinary Medicine, Conservation, and Animal Welfare, 2019. All rights reserved. Any use, in whole or in part, of the contents included in this document, including the storage, reproduction, reprocessing, dissemination or distribution of the contents through any technological platform, support or electronic network, without the prior written authorization of all the authors is prohibited.

92.	The possibility of the procedure's achievement in the scheduled planning is:	Low	Medium	High
93.	The application of the 3Rs principles in the procedure is:	Low	Medium	High
94.	Is it possible to improve the 3Rs application?	YES	NO	………………………… ……..……………………… ………………………… ……..……………………… …………………………

Ethics Laboratory for Veterinary Medicine, Conservation, and Animal Welfare, 2019. All rights reserved. Any use, in whole or in part, of the contents included in this document, including the storage, reproduction, reprocessing, dissemination or distribution of the contents through any technological platform, support or electronic network, without the prior written authorization of all the authors is prohibited.

FINAL ETHICAL ASSESSMENT OF OPU PROCEDURE			
The final assessment of section A, "DOCUMENTS", is: _____/ 13	Acceptable	Partially Acceptable	Not Acceptable
The final assessment of section B, "HARM BENEFIT EVALUATION", is: _____/ 22	Acceptable	Partially Acceptable	Not Acceptable
The final assessment of section C, "PROCEDURE PHASES QUALITY EVALUATION", is: _____/ 32	Acceptable	Partially Acceptable	Not Acceptable
The final assessment of section D, "SCIENTIFIC TEAM QUALITY EVALUATION", is: _____/ 20	Acceptable	Partially Acceptable	Not Acceptable
The final assessment of section E, "ETHICAL EVALUATION OF OPU PROCEDURE PHASES", is: _	Acceptable	Partially Acceptable	Not Acceptable
**OVERALL ETHICAL ASSESSMENT OF THE OPU PROCEDURE:**<br>*If the Ethical Assessment results for each procedure phase respects the predefined cut-off of the OPU procedure is "Acceptable"*<br>*If the Ethical Assessment results for each procedure phase does not respect the predefined cut-off of the OPU procedure is "Not acceptable"*	**Acceptable**	**Partially Acceptable**	**Not Acceptable**

Ethics Laboratory for Veterinary Medicine, Conservation, and Animal Welfare, 2019. All rights reserved. Any use, in whole or in part, of the contents included in this document, including the storage, reproduction, reprocessing, dissemination or distribution of the contents through any technological platform, support or electronic network, without the prior written authorization of all the authors is prohibited.

Comments,

Ethical B*io*R*escue* Team

Filled by

Place, Date

Signature

Ethics Laboratory for Veterinary Medicine, Conservation, and Animal Welfare, 2019. All rights reserved. Any use, in whole or in part, of the contents included in this document, including the storage, reproduction, reprocessing, dissemination or distribution of the contents through any technological platform, support or electronic network, without the prior written authorization of all the authors is prohibited.
