# Supplementary material for: An Ethical Assessment Tool (ETHAS) to Evaluate the Application of Assisted Reproductive Technologies in Mammals’ Conservation: The Case of the Northern White Rhinoceros (Ceratotherium simum cottoni)
Source: Animals (Basel). 2021 Jan 26;11(2):312. doi: 10.3390/ani11020312 (PMC7911958; doi:10.3390/ani11020312)
Supplement: Supplementary file 1 [file animals-11-00312-s001.zip › File S2.pdf]

Leibniz Institute for Zoo & Wildlife Research Berlin
BioRescue

UNIVERSITÀ DEGLI STUDI DI PADOVA

DEPARTMENT OF COMPARATIVE BIOMEDICINE AND FOOD SCIENCE

Protocol number _____________

OPU

ETHICAL RISK ASSESSMENT

Bio*Rescue* Ethical Team

Ethics Laboratory for Conservation,
Veterinary Medicine and Animal Welfare

OPU ETHICAL RISK ASSESSMENT

The Ethical Risk Assessment (ERA) allows highlighting the critical points or hazards that could occur during the execution of the OPU procedure compromising its accomplishment, and to assess the animal welfare risks. The application of ethical principles in the analysis of the risk, together with a risk ethics approach, provides a deeper analysis of the hazards and allows ethical consideration to be part of risk-related decisions. Therefore, ERA provides a base for the ethical decision-making and allows the assessment of the ethical acceptability of the procedure. For this purpose, the OPU procedure has been divided into different phases (from phase A to phase E - figure 1). Each phase has been analyzed using a detailed checklist built to identify the safety and ethical hazards and the animal welfare issues. Each item of the ERA checklist is conceptually linked and mutually integrated into an Ethical Evaluation sheet - EES (the alpha-numerical code of the first column). EES comprises the relevant ethical aspects that need to be detailed in ERA. In case of potential harms or risks, identified by the failure to reach a minimum threshold on the ERA score, corrective actions will be planned to mitigate the risks for the success of the procedure and for the animal health and welfare. The measures for the risk mitigation consist of implementing activities for reaching an acceptable fulfilment of the requirements defined in ERA or, to alleviate the adverse effects that might arise. The "as low as reasonably practicable" principle will be applied. This principle expresses that the risk should be reduced to a level that is low as reasonably practicable unless it can be demonstrated that there is a great disproportion between costs and benefits.

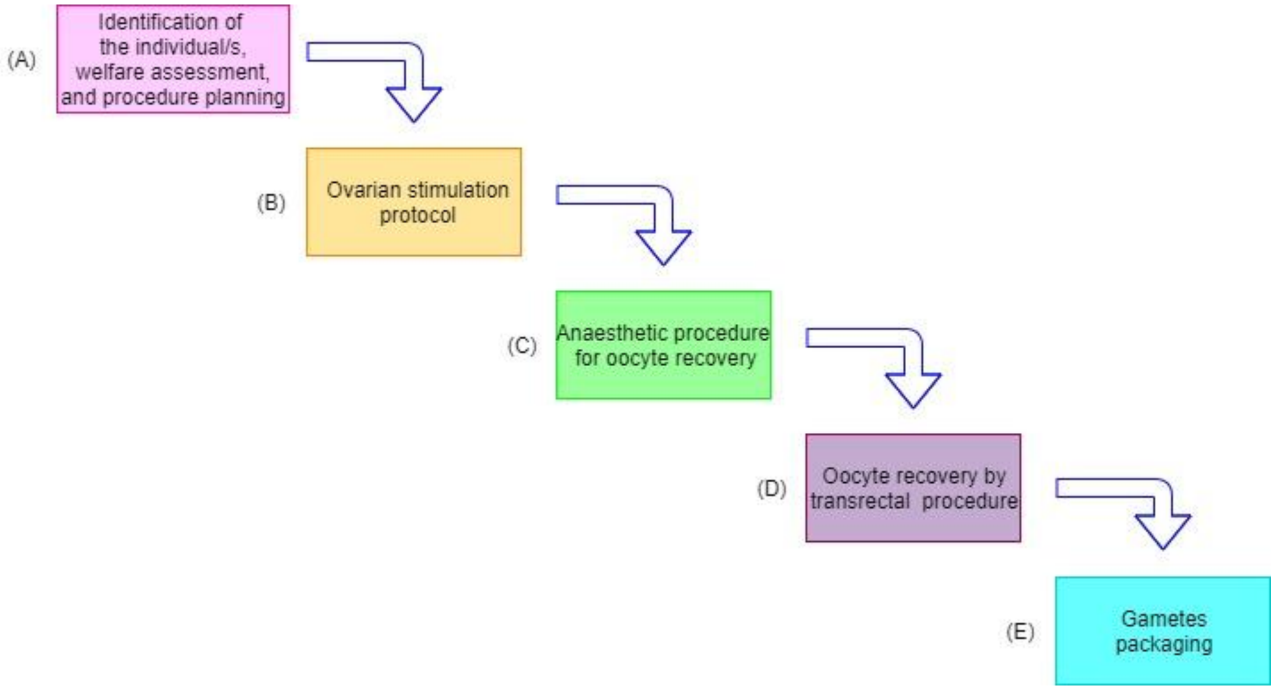

Figure 1: The flow diagram of the OPU ERA phases. The five phases (phases A-E) are shown with their relative links.

Ethics Laboratory for Veterinary Medicine, Conservation, and Animal Welfare, 2019. All rights reserved. Any use, in whole or in part, of the contents included in this document, including the storage, reproduction, reprocessing, dissemination or distribution of the contents through any technological platform, support or electronic network, without the prior written authorization of all the authors is prohibited.

Facility ________________________ Date ________________________

Procedural working area ________________________

Filled in by (*mark with an X your role and write your name*):

☐ IZW team's member: Dr. ________________________

☐ Local veterinarian: Dr. ________________________

☐ Managing Director/Responsible: Dr. ________________________

☐ Other: ________________________

PHASE A **IDENTIFICATION OF THE INDIVIDUAL/S, WELFARE ASSESSMENT AND PROCEDURE PLANNING**

EES	***Please answer, marking YES or NO, to the items in your knowledge. If an item is not relevant to you leave it blank.***	
C1	1. Has an analysis of the medical records been made to evaluate the health history of the animal/s identified for the OPU procedure?	☐ YES ☐ NO
C1	2. Has an animal inspection, in terms of body condition score, been conducted to evaluate the health of the animal/s identified for the OPU procedure?	☐ YES ☐ NO
C1	3. Has/Have the animal/s age been considered suitable to allow it to undergo the OPU procedure?	☐ YES ☐ NO
D2	4. Is there adequate diagnostic equipment available (tools necessary for the proper conduction of the general and specific physical examination, including diagnostic kits for specific procedure)?	☐ YES ☐ NO
D3	5. Are there reference laboratories (accredited laboratories with certified procedures) available to support further investigations or, at least, will they be eventually available for future procedures *in loco*?	☐ YES ☐ NO
D1	6. Do the following professionals involved or helping in OPU procedure have adequate competence to carry out an assessment of the health and welfare status of the individual/s of the species of interest?	
	a) *BioRescue* veterinarians	☐ YES ☐ NO
	b) Local veterinarians	☐ YES ☐ NO
	c) *BioRescue* staff (technicians, *etc*)	☐ YES ☐ NO
	d) Local staff (keepers, technicians, ground staff, etc.)	☐ YES ☐ NO
D1	7. To work with the species of interest, do *local veterinarians* fulfil the following requirements?	
	a) Having a licence	☐ YES ☐ NO
	b) Undergo the supervision of the national authority	☐ YES ☐ NO

Ethics Laboratory for Veterinary Medicine, Conservation, and Animal Welfare, 2019. All rights reserved. Any use, in whole or in part, of the contents included in this document, including the storage, reproduction, reprocessing, dissemination or distribution of the contents through any technological platform, support or electronic network, without the prior written authorization of all the authors is prohibited.

D1	8. To work with the species of interest, do *local staff* members fulfil the following requirements?	
	a) Having a licence	☐ YES ☐ NO
	b) Undergo the supervision of the national authority	☐ YES ☐ NO
D1	9. Do the following professionals involved or helping have adequate training to work with the species involved in the procedure?	
	a) *Bio*Rescue veterinarians	☐ YES ☐ NO
	b) Local veterinarians	☐ YES ☐ NO
	c) *Bio*Rescue staff (technicians, *etc.*)	☐ YES ☐ NO
	d) Local staff (keepers, technicians, ground staff, etc.)	☐ YES ☐ NO
C1	10. Are all the measures to prevent disturbance from the following groups adequately planned?	
	a) Public	☐ YES ☐ NO
	b) Media	☐ YES ☐ NO
	c) Staff not directly involved in the procedure	☐ YES ☐ NO
C3/ B2	11. If the animal/s has/have already undergone the OPU procedure, have at least more than three months passed after the last procedure?	☐ YES ☐ NO
C1	12. Have the following aspects of the animal welfare been assessed for at least one month *before* this OPU procedure?	
	a) Behavioural assessment	☐ YES ☐ NO
	b) Physiological assessment	☐ YES ☐ NO
C1	13. Will the following aspects of animal welfare be assessed for at least two weeks *after* this OPU procedure?	
	a) Behavioural assessment	☐ YES ☐ NO
	b) Physiological assessment	☐ YES ☐ NO
C2	14. Have details of the a), b), c) and d) being included in the overall protocol?	
	a) Procedures	☐ YES ☐ NO
	b) Techniques	☐ YES ☐ NO
	c) Materials	☐ YES ☐ NO
	d) Care and management of the animal/s before, during, and after the procedure	☐ YES ☐ NO

Ethics Laboratory for Veterinary Medicine, Conservation, and Animal Welfare, 2019. All rights reserved. Any use, in whole or in part, of the contents included in this document, including the storage, reproduction, reprocessing, dissemination or distribution of the contents through any technological platform, support or electronic network, without the prior written authorization of all the authors is prohibited.

C1	15. Have a), b), and c) been planned?	
	a) Introductory training session for all the local staff involved before the OPU procedure	☐ YES ☐ NO
	b) Briefing session, for all the staff involved, before the beginning of OPU procedure	☐ YES ☐ NO
	c) Debriefing session, for all the staff involved, after the OPU procedure to discuss the critical aspects emerged	☐ YES ☐ NO
C2	16. In case of unforeseen critical situations has an emergency protocol been developed for every single phase of the OPU procedure?	☐ YES ☐ NO
A	17. Has an ethical approval been obtained for this procedure?	☐ YES ☐ NO
**PHASE B**	**OVARIAN STIMULATION PROTOCOL**	
C1	18. In the development of the ovarian stimulation protocol, have the aspects that could *negatively* affect the welfare of the animal/s been considered?	☐ YES ☐ NO
C1	19. In the development of the ovarian stimulation protocol, have the aspects that could *positively* affect the welfare of the animal/s been considered?	☐ YES ☐ NO
C2	20. During the ovarian stimulation protocol period, has a standardized protocol been developed to verify the correct induction of the ovulation phase?	☐ YES ☐ NO
D1	21. Do the following professionals involved during the ovarian stimulation protocol phase have adequate training and expertise (both in theoretical terms and in practical terms) to carry out the procedure?	
	a) Local veterinarians	☐ YES ☐ NO
	b) Local staff (keepers, etc.)	☐ YES ☐ NO
D2	22. Has the equipment for correct conduction of the ovarian stimulation phase and administration of the treatment been evaluated safe for animal use?	☐ YES ☐ NO
C1	23. Will the following aspects of animal welfare be assessed during the ovarian stimulation protocol phase?	
	a) Behavioural assessment	☐ YES ☐ NO
	b) Physiological assessment	☐ YES ☐ NO

Ethics Laboratory for Veterinary Medicine, Conservation, and Animal Welfare, 2019. All rights reserved. Any use, in whole or in part, of the contents included in this document, including the storage, reproduction, reprocessing, dissemination or distribution of the contents through any technological platform, support or electronic network, without the prior written authorization of all the authors is prohibited.

PHASE C	ANAESTHETIC PROCEDURE FOR OOCYTE RECOVERY	
C1	24. Has a detailed animal pre-anaesthetic medical assessment been conducted?	☐ YES ☐ NO
C3	25. Have the following aspects in anaesthesia drug administration been considered in order to create less stress for the individual/s and provide greater control of drug induction?	
	a) Training procedures	☐ YES ☐ NO
	b) Restraining area	☐ YES ☐ NO
C1	26. Have specific protocols been developed to control the correct execution of a), b), c) and d)?	
	a) Pre-anaesthetic visit	☐ YES ☐ NO
	b) Drug induction	☐ YES ☐ NO
	c) Animal health monitoring	☐ YES ☐ NO
	d) Recovery	☐ YES ☐ NO
C1	27. Have specific protocols/tools been developed to verify if all the needed equipment is available and correctly operating for the anaesthetic procedure phase?	☐ YES ☐ NO
D2	28. Have specific protocols/tools for the anaesthetic procedure phase been developed to verify if all the needed drugs are available?	☐ YES ☐ NO
D3	29. Is the anaesthetic procedure place suitable (in terms of light, temperature, aeration, hygiene, and cleaning) for a) and b)?	
	a) Animal safety	☐ YES ☐ NO
	b) Staff safety	☐ YES ☐ NO
C3	30. Have protocols and/or actions to minimize animal's suffering, both during and after the anaesthetic phase, been developed?	☐ YES ☐ NO
C3/ C2	31. Is there a cut-off time for the anaesthetic phase?	☐ YES ☐ NO
C3/ D2	32. Has the equipment for correct conduction of the anaesthetic phase, administration of the treatment, and the animal health monitoring been evaluated safe for animal use?	☐ YES ☐ NO
D1	33. Do the following professionals involved or helping during the anaesthetic phase have adequate training and expertise (both in theoretical terms and in practical terms) to carry out the procedure?	
	a) *BioRescue* veterinarians	☐ YES ☐ NO
	b) Local veterinarians	☐ YES ☐ NO
	c) *BioRescue* staff (technicians, etc.)	☐ YES ☐ NO
	d) Local staff (keepers, technicians, ground staff, etc.)	☐ YES ☐ NO

Ethics Laboratory for Veterinary Medicine, Conservation, and Animal Welfare, 2019. All rights reserved. Any use, in whole or in part, of the contents included in this document, including the storage, reproduction, reprocessing, dissemination or distribution of the contents through any technological platform, support or electronic network, without the prior written authorization of all the authors is prohibited.

PHASE D	OOCYTE RECOVERY BY TRANSRECTAL PROCEDURE	
C3/ D2	34. Is there adequate equipment for the monitoring of the animal/s during this phase (in terms of measuring heart rate, blood sampling, clinical observation)?	☐ YES ☐ NO
C1/ D2	35. Have protocols for the sterilization and sanitation been developed and optimized for the following aspects?	
	a) Equipment for the procedure	☐ YES ☐ NO
	b) Laboratory reagents and medication	☐ YES ☐ NO
	c) Personal protective equipment (PPE)	☐ YES ☐ NO
D2	36. Have protocols been developed to verify the following aspects for the OPU procedure phase?	
	a) Availability of the equipment	☐ YES ☐ NO
	b) Equipment operativity	☐ YES ☐ NO
C1	37. Is there any specific protocol to reduce infection risks related to the procedures of this phase?	☐ YES ☐ NO
C1	38. Before starting the OPU procedure phase, has the availability of personal protective equipment (PPE) for all the staff members involved been checked?	☐ YES ☐ NO
D	39. Is the area where the oocytes recovery take place suitable (in terms of light, temperature, aeration, hygiene, and cleaning) for a) and b)?	
	a) Animal safety	☐ YES ☐ NO
	b) Staff safety	☐ YES ☐ NO
C3	40. Is the method used the one that causes the least pain, suffering, distress or lasting harm and the one that provides the best satisfactory results?	
C1	41. In the development of the oocytes collection protocol, have the following aspects that could affect the a) and b), either during and after the procedure phase, been considered?	
	a) Animal health	☐ YES ☐ NO
	b) Animal suffering	☐ YES ☐ NO
C3	42. In case of unforeseen emergency situations, during the oocytes recovery phase, where the animal/s cannot be treated and suffer/s severely, has euthanasia intervention been planned?	☐ YES ☐ NO
C1	43. To evaluate the quality of oocytes obtained, has a gametes grade of scoring been established?	☐ YES ☐ NO
C1	44. Is the oocytes conservation medium the best, based on the present scientific knowledge, for rhinos' cells?	☐ YES ☐ NO

☐
☐

Ethics Laboratory for Veterinary Medicine, Conservation, and Animal Welfare, 2019. All rights reserved. Any use, in whole or in part, of the contents included in this document, including the storage, reproduction, reprocessing, dissemination or distribution of the contents through any technological platform, support or electronic network, without the prior written authorization of all the authors is prohibited.

D1	45. Do the following professionals involved or helping during the oocytes recovery phase have adequate training and expertise (both in theoretical terms and in practical terms) to carry out the procedure?	
	c) *BioRescue* veterinarians	☐ YES ☐ NO
	d) Local veterinarians	☐ YES ☐ NO
	e) *BioRescue* staff (technicians, etc.)	☐ YES ☐ NO
	f) Local staff (keepers, technicians, ground staff, etc.)	☐ YES ☐ NO
**PHASE E**	**GAMETES PACKAGING**	
D2	46. Has a protocol/tool been developed to verify if all the needed equipment is available and correctly operating for this phase?	☐ YES ☐ NO
D2	47. Have protocols for the sterilization and sanitation been developed and optimized for a), b), and c)?	
	a) Equipment for the procedure	☐ YES ☐ NO
	b) Laboratory reagents and medication	☐ YES ☐ NO
	c) Personnel personal protective equipment (PPE)?	☐ YES ☐ NO
C1/ D2	48. Has a protocol/tool been developed to verify if all the necessary materials (flushing media, etc) are available?	☐ YES ☐ NO
D2	49. Has a standardized protocol been developed for labelling and marking the specimens for the correct identification and classification?	☐ YES ☐ NO
D3	50. Is the area where the oocytes analysis and labelling take place suitable (in terms of light, temperature, aeration, hygiene, and cleaning) for a) and b)?	
	a) Cell safety?	☐ YES ☐ NO
	b) Staff safety?	☐ YES ☐ NO
D2	51. Are the specimens transported into a portable device that allows controlling the adequate temperature chain?	☐ YES ☐ NO
D2	52. Is the portable device been checked for the following aspects?	
	a) Operativity	☐ YES ☐ NO
	b) Sterilization/sanitation	☐ YES ☐ NO

Ethics Laboratory for Veterinary Medicine, Conservation, and Animal Welfare, 2019. All rights reserved. Any use, in whole or in part, of the contents included in this document, including the storage, reproduction, reprocessing, dissemination or distribution of the contents through any technological platform, support or electronic network, without the prior written authorization of all the authors is prohibited.

Comments,

I hereby give my consent for the processing of data provided on the Ethical Risk Analysis (ERA) form to be stored, processed, analyzed and published by B*io*R*escue* project partner for scientific research purposes.

Place, Date

Signature

Ethics Laboratory for Veterinary Medicine, Conservation, and Animal Welfare, 2019. All rights reserved. Any use, in whole or in part, of the contents included in this document, including the storage, reproduction, reprocessing, dissemination or distribution of the contents through any technological platform, support or electronic network, without the prior written authorization of all the authors is prohibited.
