# Supplementary material for: An Ethical Assessment Tool (ETHAS) to Evaluate the Application of Assisted Reproductive Technologies in Mammals’ Conservation: The Case of the Northern White Rhinoceros (Ceratotherium simum cottoni)
Source: Animals (Basel). 2021 Jan 26;11(2):312. doi: 10.3390/ani11020312 (PMC7911958; doi:10.3390/ani11020312)
Supplement: Supplementary file 1 [file animals-11-00312-s001.zip › File S3.pdf]

Leibniz Institute for Zoo & Wildlife Research Berlin

BioRescue

Università degli Studi di Padova

Department of Comparative Biomedicine and Food Science

IVF Laboratory Procedures

ETHICAL EVALUATION SHEET

Bio*Rescue* Ethical Team

Ethics Laboratory for Conservation,
Veterinary Medicine and Animal Welfare

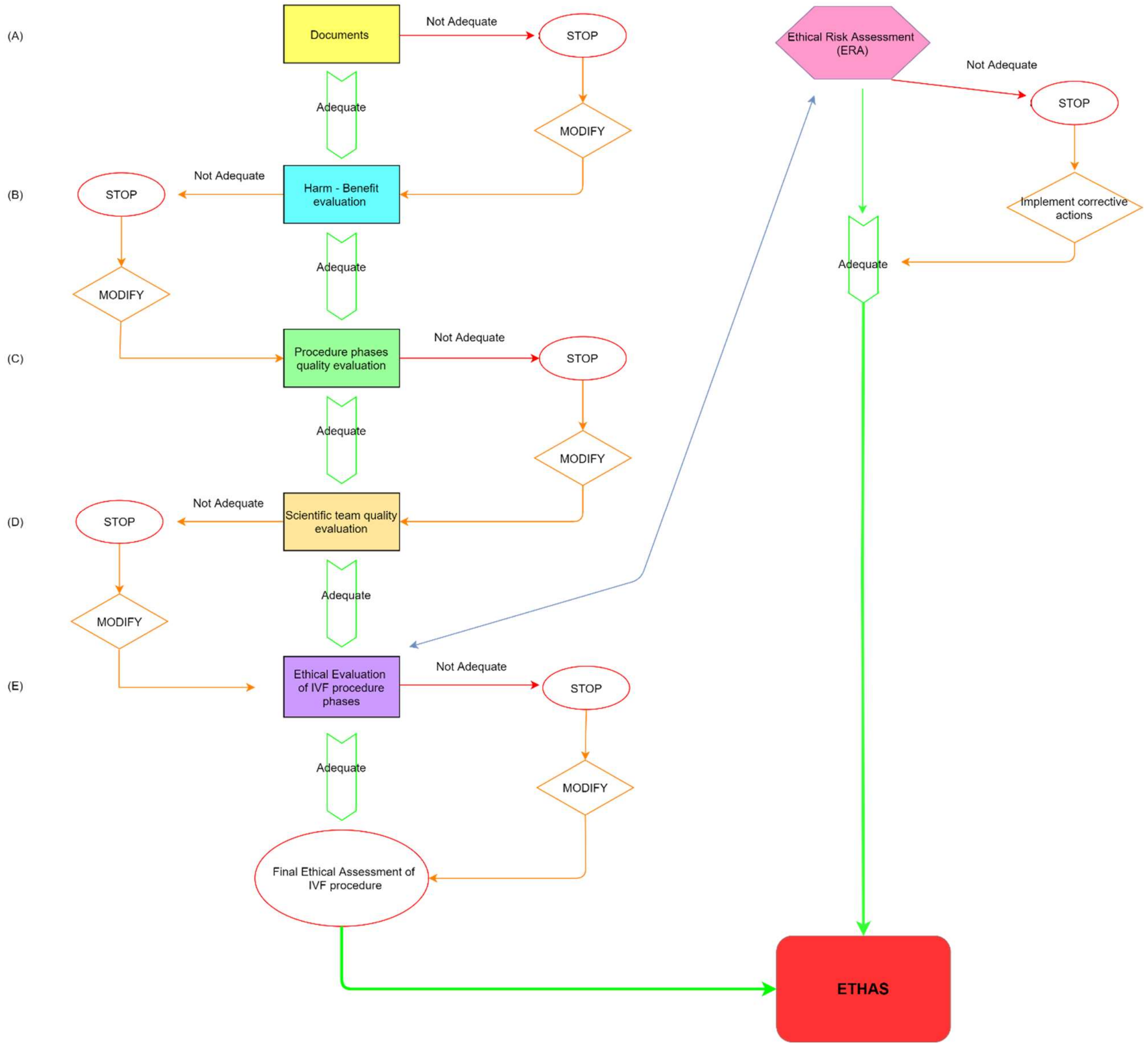

Figure 1: Flow chart for IVF phases (from A to E).

Ethics Laboratory for Veterinary Medicine, Conservation, and Animal Welfare, 2019. All rights reserved. Any use, in whole or in part, of the contents included in this document, including the storage, reproduction, reprocessing, dissemination or distribution of the contents through any technological platform, support or electronic network, without the prior written authorization of all the authors is prohibited.

A	DOCUMENTS			
				Details when needed
1.	Has the IVF protocol been planned in compliance with the Directive 2010/63/EU?	YES	NO	………………………… ………..……………… ………………………… ……………..……… …………………………
2.	When a non-EU country is involved, do the research-related activities, undertaken in that country, raise potential ethics issues?<br><br>*Horizon 2020: Being outside the reach of European laws and standards, such procedure can raise specific ethical issues (particularly in developing countries): "exploitation of participants, exploitation of local resources, risks to workers & staff".*<br><br>If yes, specify<br>……………………………………………………………… ……………………………………………………………… ………………………………………………………	YES	NO	………………………… ………..……………… ………………………… ……………..……… …………………………
3.	If animal specimens of low and/or lower-middle-income countries are involved, have documents showing compliance with the following normative been submitted to the competent authority:			………………………… ………..……………… ………………………… ……………..……… …………………………
	a) Nagoya Protocol on access and benefit-sharing?	YES	NO	
	b) EU Regulation No 511/2014 (on compliance of Nagoya Protocol)?	YES	NO	
	Horizon 2020: "Resources from a non-EU country — Any use of local resources (especially animal and/or human tissue samples, genetic material, live animals, human remains, materials of historical value, endangered fauna or flora samples, fossils) must show respect for cultural traditions and share benefits (i.e. also benefit local participants and their communities, involve local workers – as equal partners – and respond to local worker's needs)."			

Ethics Laboratory for Veterinary Medicine, Conservation, and Animal Welfare, 2019. All rights reserved. Any use, in whole or in part, of the contents included in this document, including the storage, reproduction, reprocessing, dissemination or distribution of the contents through any technological platform, support or electronic network, without the prior written authorization of all the authors is prohibited.

4.	If the IVF procedures are part of a project that involves low and/or lower-middle-income countries, are benefit-sharing measures been planned?<br>Please, give details on:<br>a) Benefit sharing measures.<br>b) Responsiveness to local population needs.<br>a) ………………………………………………………… …………………………………………………………<br>b) ………………………………………………………… …………………………………………………………	YES	NO	……………………… ……………………… ……………………… ……………………… ………………………
5.	If needed, have export licenses, to import biomaterial from non-EU countries into the EU, been obtained?<br>*Horizon 2020 "Import/export of material — If genetic resources are transferred across borders, it may be mandatory under the law of the provider country to obtain an authorisation for the transfer. In addition, you must use an agreement which describes the conditions for the export and the terms of utilisation and, if applicable, relevant benefit-sharing measures."*	YES	NO	……………………… ……………………… ……………………… ……………………… ………………………
6.	Do the laboratories involved have an ISO accreditation?<br>If yes, give details.<br>……………………………………………………………	YES	NO	……………………… ……………………… ……………………… ……………………… ………………………
7.	Has an ethical approval been obtained?<br>If yes, give details.<br>……………………………………………………………	YES	NO	……………………… ……………………… ……………………… ……………………… ………………………
8.	Have the licenses to the practice of all the staff involved in the IVF procedures been submitted?	YES	NO	……………………… ……………………… ……………………… ……………………… ………………………
9.	Have certificates of good standing been submitted?<br>*(e.g. a certification that no measures of disciplinary action of professional or administrative nature had been taken for all the staff involved in the procedure)*	YES	NO	……………………… ……………………… ……………………… ……………………… ………………………

Ethics Laboratory for Veterinary Medicine, Conservation, and Animal Welfare, 2019. All rights reserved. Any use, in whole or in part, of the contents included in this document, including the storage, reproduction, reprocessing, dissemination or distribution of the contents through any technological platform, support or electronic network, without the prior written authorization of all the authors is prohibited.

B	HARM - BENEFIT EVALUATION			
B1	Benefit evaluation			
				Details when needed
10.	Are the IVF procedures part of a novel wildlife conservation strategy?	YES	NO	………………………………………………………………………………………………
11.	Will the application of the IVF procedures improve efforts and the advancement of reproductive scientific knowledge for developing new conservation strategies?	YES	NO	………………………………………………………………………………………………
12.	Could the effects of the IVF procedure, as part of a novel conservation strategy, have a positive impact on the natural habitat in the future?	YES	NO	………………………………………………………………………………………………
13.	Will the scientific improvements obtained from the IVF procedures impact positively on other scientific fields?	YES	NO	………………………………………………………………………………………………
14.	Will the application of the IVF procedures improve the professional training of the laboratory personnel?<br>If yes, please give details<br>………………………………………………………………………………………	YES	NO	………………………………………………………………………………………………
15.	Is the scientific knowledge achievable from IVF procedure optimization/application relevant to the assisted reproductive technology (ART) for mammals?<br>If yes, please give details<br>………………………………………………………………………………………	YES	NO	………………………………………………………………………………………………

Ethics Laboratory for Veterinary Medicine, Conservation, and Animal Welfare, 2019. All rights reserved. Any use, in whole or in part, of the contents included in this document, including the storage, reproduction, reprocessing, dissemination or distribution of the contents through any technological platform, support or electronic network, without the prior written authorization of all the authors is prohibited.

16.	Could the IVF procedures or part of them have positive side effects on the species involved?<br>If yes, which ones?<br>……………………………………………………………<br>……………………………………………………………<br>……………………………………………………………	YES	NO	………………………<br>………………………<br>………………………<br>………………………<br>………………………
**B2**	**Harm evaluation**			
				Details when needed
17.	Could these IVF procedures or part of them have adverse side effects on the health of the species involved?<br>If yes, which ones?<br>……………………………………………………………<br>……………………………………………………………<br>……………………………………………………………	YES	NO	………………………<br>………………………<br>………………………<br>………………………<br>………………………
18.	Have the possible adverse effects on the specimens, during the following steps, been considered?			………………………<br>………………………<br>………………………<br>………………………<br>………………………
	a) Oocytes maturation	YES	NO	
	b) Sperm recovery	YES	NO	
	c) Gametes preparation for IVF	YES	NO	
	d) Embryos developement	YES	NO	
	e) Specimens long-term cryopreservation	YES	NO	

Ethics Laboratory for Veterinary Medicine, Conservation, and Animal Welfare, 2019. All rights reserved. Any use, in whole or in part, of the contents included in this document, including the storage, reproduction, reprocessing, dissemination or distribution of the contents through any technological platform, support or electronic network, without the prior written authorization of all the authors is prohibited.

	If yes, give details<br><br>a) ………………………………………………………<br>b) ………………………………………………………<br>c) ………………………………………………………<br>d) ………………………………………………………<br>e) ………………………………………………………			
19.	Have the possible negative effects (public opinions, scientific opinions, etc.), that may occur to the biological specimens of the northern white rhinoceros during the IVF procedures, been considered and discussed with the project partners?	YES	NO	………………………<br>………………………<br>………………………<br>………………………<br>………………………
20.	Is there any other concern about the harm evaluation of the IVF procedure you would like to underline?<br><br>If yes, give details<br>……………………………………………………………<br>……………………………………………………………<br>……………………………………………………………	YES	NO	………………………<br>………………………<br>………………………<br>………………………<br>………………………

Ethics Laboratory for Veterinary Medicine, Conservation, and Animal Welfare, 2019. All rights reserved. Any use, in whole or in part, of the contents included in this document, including the storage, reproduction, reprocessing, dissemination or distribution of the contents through any technological platform, support or electronic network, without the prior written authorization of all the authors is prohibited.

C	PROCEDURE PHASES QUALITY EVALUATION			
**C1**	**Pre-screening considerations**			
				Details when needed
21.	Has the quality of the innovative IVF procedure or the equipment been already tested in other related studies?<br><br>If yes, give details<br>……………………………………………………………<br>……………………………………………………………<br>……………………………………………………………	YES	NO	……………………… ……………………… ……………………… ……………………… ………………………
22.	Since IVF procedure is commonly used in farm and domestic animals, have the necessary species-specific adaptations being considered?	YES	NO	……………………… ……………………… ……………………… ……………………… ………………………
23.	Has the opportunity to modify any step of the IVF protocol ever been considered to improve the procedure for the rhinoceros' specimens?	YES	NO	……………………… ……………………… ……………………… ……………………… ………………………
24.	Has an IVF procedural timetable for each single phases of the IVF laboratory procedures been drawn up?	YES	NO	……………………… ……………………… ……………………… ……………………… ………………………
25.	Are the project partners committed to eventually publishing also critical events so that they could be avoided in future projects?<br><br>If no, explain why.<br>……………………………………………………………<br>……………………………………………………………<br>……………………………………………………………	YES	NO	……………………… ……………………… ……………………… ……………………… ………………………
26.	Has it been planned to share and publish relevant data of procedures refinement?<br><br>If no, explain why.<br>……………………………………………………………<br>……………………………………………………………<br>……………………………………………………………	YES	NO	……………………… ……………………… ……………………… ……………………… ………………………

Ethics Laboratory for Veterinary Medicine, Conservation, and Animal Welfare, 2019. All rights reserved. Any use, in whole or in part, of the contents included in this document, including the storage, reproduction, reprocessing, dissemination or distribution of the contents through any technological platform, support or electronic network, without the prior written authorization of all the authors is prohibited.

**C2**	**Procedural steps evaluation**			
				Details when needed
27.	Have the sperm collection and recovery protocols been tested and evaluated in other related species?	YES	NO	……………………… ……………………… ……………………… ……………………… ………………………
28.	Has the oocytes maturation protocol been tested and evaluated in other related species?	YES	NO	……………………… ……………………… ……………………… ……………………… ………………………
29.	Has the oocytes ICSI protocol been tested and evaluated in other related species?	YES	NO	……………………… ……………………… ……………………… ……………………… ………………………
30.	Have the embryo development protocol been tested and evaluated in other related species?	YES	NO	……………………… ……………………… ……………………… ……………………… ………………………
31.	Has the method used to select the spermatozoa that will be used for the ICSI been optimized in other related species?	YES	NO	……………………… ……………………… ……………………… ……………………… ………………………
**C3**	**3Rs evaluation**			
Replacement				
				Details when needed
32.	Have alternative procedural steps or parts of them been considered for the execution of the project?<br>If not, why?<br>If yes, explain.<br>……………………………………………………………… ………………………………………………………………	YES	NO	……………………… ……………………… ……………………… ……………………… ………………………
33.	Has the possibility to change some procedural steps for an incomplete replacement been considered?<br>If yes, give some details.<br>……………………………………………………………… ………………………………………………………………	YES	NO	……………………… ……………………… ……………………… ……………………… ………………………

Ethics Laboratory for Veterinary Medicine, Conservation, and Animal Welfare, 2019. All rights reserved. Any use, in whole or in part, of the contents included in this document, including the storage, reproduction, reprocessing, dissemination or distribution of the contents through any technological platform, support or electronic network, without the prior written authorization of all the authors is prohibited.

34.	Is it possible to replace the medium extracted from other animals with synthetic ones? (e.g. *rhinoceros estrum serum, BSA*, etc.)	YES	NO	....
35.	Is it possible to optimize the procedures on other species not endangered?	YES	NO	....
36.	Have other cryopreservation methodologies been evaluated for a more efficient sperm long-term stocking?	YES	NO	....
Reduction				
37.	Have IVF procedure phases been optimized so that a minimum number of collected specimens will be used?	YES	NO	....
38.	Will the gametes or embryos be shared with other scientific groups?<br>If yes, give details.<br>If no, give details.<br>..	YES	NO	....
39.	Is it possible to thaw the minimum necessary aliquot of sperm?	YES	NO	....
40.	Could the specimens that cannot be used for the IVF procedure (e.g., surplus of spermatozoa, oocytes and embryos not adequately developed) be used for other studies?	YES	NO	....

Ethics Laboratory for Veterinary Medicine, Conservation, and Animal Welfare, 2019. All rights reserved. Any use, in whole or in part, of the contents included in this document, including the storage, reproduction, reprocessing, dissemination or distribution of the contents through any technological platform, support or electronic network, without the prior written authorization of all the authors is prohibited.

Refinement				
41.	Have IVF laboratory procedures ever been applied to other animal specimens of other species, so that any possible risk has already been evaluated, analysed and minimized?	YES	NO	……………………… ……………………… ……………………… ……………………… ………………………
42.	Have alternative procedural steps or part of them that might reduce possible cellular stress of the following specimens been considered?			……………………… ……………………… ……………………… ……………………… ………………………
	a) Oocytes	YES	NO	
	b) Spermatozoa	YES	NO	
	c) Embryos	YES	NO	
43.	Have mitigation actions been included in the protocols to reduce the possible adverse effects that the following specimens might encounter? (i.e. chemical and physical parameters control, *etc*.)			……………………… ……………………… ……………………… ……………………… ………………………
	a) Oocytes maturation	YES	NO	
	b) Sperm recovery	YES	NO	
	c) Gametes preparation for IVF	YES	NO	
	d) Embryos development	YES	NO	
	e) Specimens long-term cryopreservation	YES	NO	
44.	To avoid cellular stress, during the IVF procedure, have the external variables (chemicals and physical parameters) been kept within adequate parameters?	YES	NO	……………………… ……………………… ……………………… ……………………… ………………………
45.	Is it possible to optimize the following procedures?			……………………… ……………………… ……………………… ……………………… ………………………
	a) maturation oocyte	YES	NO	
	b) sperm recovery	YES	NO	

Ethics Laboratory for Veterinary Medicine, Conservation, and Animal Welfare, 2019. All rights reserved. Any use, in whole or in part, of the contents included in this document, including the storage, reproduction, reprocessing, dissemination or distribution of the contents through any technological platform, support or electronic network, without the prior written authorization of all the authors is prohibited.

D	SCIENTIFIC TEAM QUALITY EVALUATION			
**D1**	**Team and Teamwork**			
				Details when needed
46.	Are the team's economic resources adequate for the following a) and b) situations?<br>a) all the project<br>b) to overcome problematic situations or emergency management in case of necessity	YES	NO	………………………<br>………………………<br>………………………<br>………………………<br>………………………
47.	Has the team already conducted IVF procedures on animals related to the sub-species involved?	YES	NO	………………………<br>………………………<br>………………………<br>………………………<br>………………………
48.	Has the team already taken part in relevant and innovative reproductive procedures?	YES	NO	………………………<br>………………………<br>………………………<br>………………………<br>………………………
49.	Has the team already developed innovative methodologies?	YES	NO	………………………<br>………………………<br>………………………<br>………………………<br>………………………
50.	Is someone deciding the team members for the IVF procedure?<br>If yes, give details.<br>……………………………………………………<br>……………………………………………………<br>……………………………………………………	YES	NO	………………………<br>………………………<br>………………………<br>………………………<br>………………………
51.	Is someone responsible for all the IVF procedure phases?<br>If yes, give details.<br>……………………………………………………<br>……………………………………………………<br>……………………………………………………	YES	NO	………………………<br>………………………<br>………………………<br>………………………<br>………………………
52.	Do all the staff members have defined roles and clear responsibilities in the IVF procedure?	YES	NO	………………………<br>………………………<br>………………………<br>………………………<br>………………………

Ethics Laboratory for Veterinary Medicine, Conservation, and Animal Welfare, 2019. All rights reserved. Any use, in whole or in part, of the contents included in this document, including the storage, reproduction, reprocessing, dissemination or distribution of the contents through any technological platform, support or electronic network, without the prior written authorization of all the authors is prohibited.

53.	Are all the staff members involved in the IVF experienced in this procedure?	YES	NO	...........................<br>...........................<br>...........................<br>...........................<br>...........................
54.	Are all the staff members, involved at any level in the IVF procedure, adequately educated, competent and continuously trained and supervised until they have demonstrated the required competence?	YES	NO	...........................<br>...........................<br>...........................<br>...........................<br>...........................
55.	Has a person been designed to monitor the specimen's conservation status during the long-term cryopreservation procedure?	YES	NO	...........................<br>...........................<br>...........................<br>...........................<br>...........................
56.	Is someone responsible for the following steps been assigned?			...........................<br>...........................<br>...........................<br>...........................<br>...........................
	a) stocking of the biological material	YES	NO	
	b) transport of the biological material	YES	NO	
	c) transport of embryos from the IVF laboratory to the embryo transfer place	YES	NO	
57.	Is the staff member in charge of the transport of the following specimens trained for biological material transportation?			...........................<br>...........................<br>...........................<br>...........................<br>...........................
	a) oocytes	YES	NO	
	b) spermatozoa	YES	NO	
	c) embryos	YES	NO	
**D2**	**Equipment**			
				Details when needed
58.	Is the adequate equipment, necessary for the proper conduction of the all IVF procedure phases, available?	YES	NO	...........................<br>...........................<br>...........................<br>...........................<br>...........................
59.	Is the equipment adequate for the specimens (gametes and embryos) used for the IVF procedure?	YES	NO	...........................<br>...........................<br>...........................<br>...........................<br>...........................

Ethics Laboratory for Veterinary Medicine, Conservation, and Animal Welfare, 2019. All rights reserved. Any use, in whole or in part, of the contents included in this document, including the storage, reproduction, reprocessing, dissemination or distribution of the contents through any technological platform, support or electronic network, without the prior written authorization of all the authors is prohibited.

60.	Before starting the procedure, has the availability of personal protective equipment (PPE) for all the staff members involved been checked?	YES	NO	…………………………………………………………………………………………
61.	Has a control checklist been developed to verify if all the equipment needed for the procedure are available and correctly operating and, if needed, sterilized?	YES	NO	…………………………………………………………………………………………
**D3**	**Laboratories and BioBanks**			
				Details when needed
62.	Are specimens correctly processed in the BioBank?	YES	NO	…………………………………………………………………………………………
63.	Has a Risk Assessment of all the laboratory activities been performed?	YES	NO	…………………………………………………………………………………………
64.	If possible, are specimens divided into aliquots and stored in more than one laboratory?	YES	NO	…………………………………………………………………………………………

Ethics Laboratory for Veterinary Medicine, Conservation, and Animal Welfare, 2019. All rights reserved. Any use, in whole or in part, of the contents included in this document, including the storage, reproduction, reprocessing, dissemination or distribution of the contents through any technological platform, support or electronic network, without the prior written authorization of all the authors is prohibited.

E	ETHICAL EVALUATION OF IVF-lab PROCEDURE PHASES			
				Details when needed
65.	Has an IVF-lab Ethical Risk Assessment been planned for each laboratory involved in the procedure?	YES	NO	………
66.	In case of a non-satisfied requirement, emerging from the Ethical Risk Assessment, will risk mitigation actions be implemented?	YES	NO	………
67.	Does the procedure accomplish with all the International and National Regulations?	YES	NO	………
68.	Has the staff safety been adequately evaluated with dedicated items in the IVF-lab Ethical Risk Assessment?	YES	NO	………
69.	The benefits deriving from the procedures' application are:	Low		Medium / High
70.	The possibility of the procedures' successful accomplishment is:	Low		Medium / High
71.	The possibility of the procedures' achievement in the scheduled planning is:	Low		Medium / High
72.	The application of the 3Rs principles in the procedures is:	Low		Medium / High
73.	Is it possible to improve the application of the 3Rs?	YES	NO	………

Ethics Laboratory for Veterinary Medicine, Conservation, and Animal Welfare, 2019. All rights reserved. Any use, in whole or in part, of the contents included in this document, including the storage, reproduction, reprocessing, dissemination or distribution of the contents through any technological platform, support or electronic network, without the prior written authorization of all the authors is prohibited.

FINAL ETHICAL ASSESSMENT OF IVF-lab PROCEDURE			
The final assessment of section A, "DOCUMENTS", is: _____/ 10	Acceptable	Partially Acceptable	Not Acceptable
The final assessment of section B, "HARM BENEFIT EVALUATION", is: _____/ 15	Acceptable	Partially Acceptable	Not Acceptable
The final assessment of section C, "PROCEDURE PHASES QUALITY EVALUATION", is: _____/ 32	Acceptable	Partially Acceptable	Not Acceptable
The final assessment of section D, "SCIENTIFIC TEAM QUALITY EVALUATION", is: _____/ 24	Acceptable	Partially Acceptable	Not Acceptable
The final assessment of section E, "ETHICAL EVALUATION OF IVF PROCEDURE PHASES", is:	Acceptable	Partially Acceptable	Not Acceptable
**OVERALL ETHICAL ACCEPTABILITY OF IVF PROCEDURE:**<br>*If the Ethical Assessment results for each procedure phase respects the predefined cut-off of IVF procedure is "Acceptable"*<br>*If the Ethical Assessment results for each procedure phase does not respect the predefined cut-off of IVF procedure is "Not acceptable"*	**Acceptable**	**Partially Acceptable**	**Not Acceptable**

Ethics Laboratory for Veterinary Medicine, Conservation, and Animal Welfare, 2019. All rights reserved. Any use, in whole or in part, of the contents included in this document, including the storage, reproduction, reprocessing, dissemination or distribution of the contents through any technological platform, support or electronic network, without the prior written authorization of all the authors is prohibited.

Comments,

Ethical B*io*R*escue* Team

Filled by

Place, Date

Signature

Ethics Laboratory for Veterinary Medicine, Conservation, and Animal Welfare, 2019. All rights reserved. Any use, in whole or in part, of the contents included in this document, including the storage, reproduction, reprocessing, dissemination or distribution of the contents through any technological platform, support or electronic network, without the prior written authorization of all the authors is prohibited.
