# Supplementary material for: An Ethical Assessment Tool (ETHAS) to Evaluate the Application of Assisted Reproductive Technologies in Mammals’ Conservation: The Case of the Northern White Rhinoceros (Ceratotherium simum cottoni)
Source: Animals (Basel). 2021 Jan 26;11(2):312. doi: 10.3390/ani11020312 (PMC7911958; doi:10.3390/ani11020312)
Supplement: Supplementary file 1 [file animals-11-00312-s001.zip › File S4.pdf]

Leibniz Institute for Zoo & Wildlife Research Berlin

BioRescue

UNIVERSITÀ DEGLI STUDI DI PADOVA

DEPARTMENT OF COMPARATIVE BIOMEDICINE AND FOOD SCIENCE

Protocol number ____________

IVF Laboratory procedures

ETHICAL RISK ASSESSMENT

Bio*Rescue* Ethical Team

Ethics Laboratory for Conservation,
Veterinary Medicine and Animal Welfare

ETHICAL RISK ASSESSMENT

The Ethical Risk Assessment (ERA) allows highlighting the critical points or hazards that could occur during the execution of IVF Laboratory (IVF-lab) Procedures compromising their accomplishment. The application of ethical principles in the analysis of the risk, together with a risk ethics approach, provides a deeper analysis of the hazards and allows ethical consideration to be part of risk-related decisions. Therefore, ERA provides a base for the ethical decision-making and allows the assessment of the ethical acceptability of the procedure. For this purpose, the IVF Laboratory Procedures have been divided into different phases (from phase A to phase I - figure 1). Each phase has been analyzed using a detailed checklist built to identify the safety for the specimens and workers and ethical hazards. Each item of the ERA checklist is conceptually linked and mutually integrated into an Ethical Evaluation Sheet - EES (the alpha-numerical code of the first column). EES comprises the relevant ethical aspects that need to be detailed in ERA. In case of potential harms or risks, identified by the failure to reach a minimum threshold on the ERA score, corrective actions will be planned to mitigate the risks for the success of the procedures. The measures for the risk mitigation consist of implementing activities for reaching an acceptable fulfilment of the requirements defined in ERA or to alleviate the adverse effects that might arise. The "as low as reasonably practicable" principle will be applied. This principle expresses that the risk should be reduced to a level that is low as reasonably practicable unless it can be demonstrated that there is a great disproportion between the cost and the benefits.

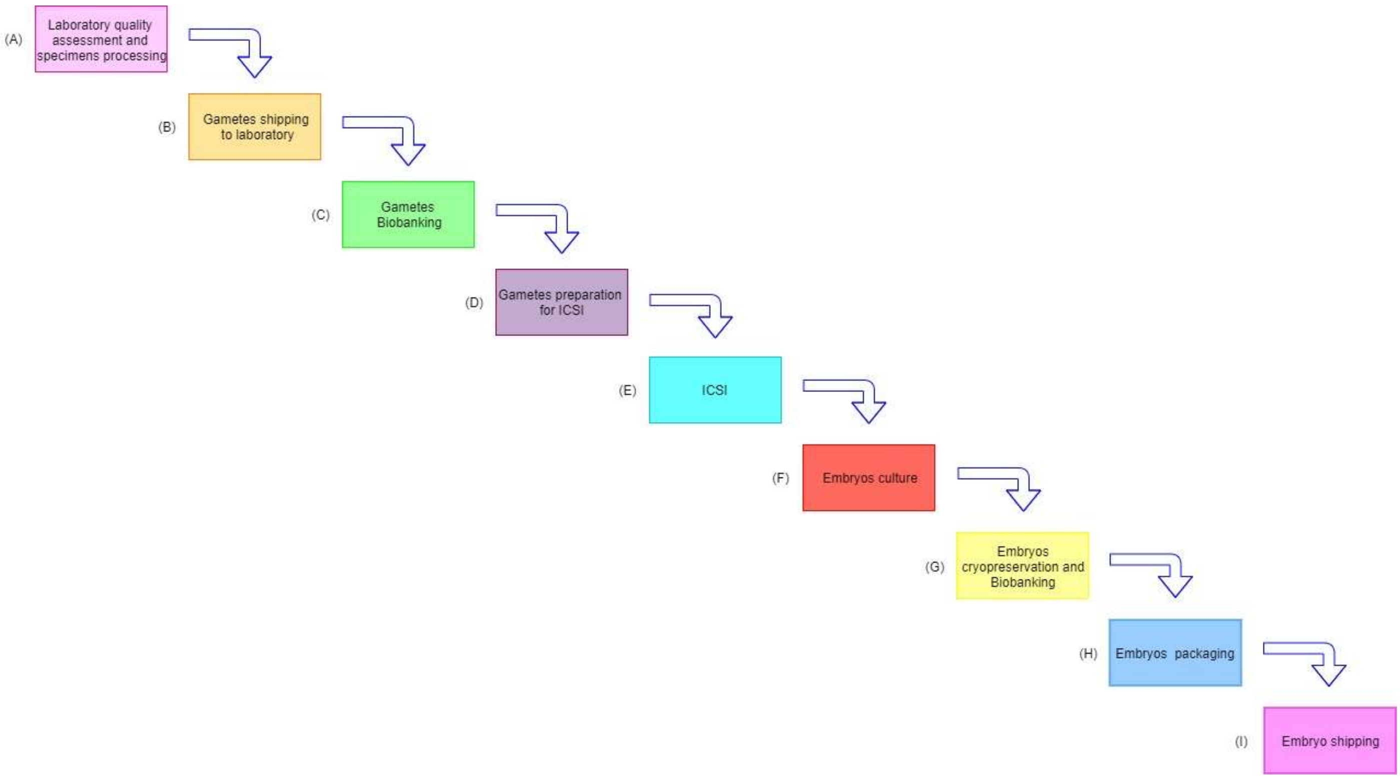

Figure 1: The flow diagram of the IVF Laboratory Procedures ERA phases. The nine phases (phases A-I) are shown with their relative links.

Laboratory ______________________ Date ______________________

Filled by ______________________

PHASE A LABORATORY QUALITY ASSESSMENT AND SPECIMENS PROCESSING

EES	***Please answer, marking YES or NO, to the items in your knowledge. If an item is not relevant to you leave it blank.***	
A	1. Does the laboratory have an ISO accreditation?<br><br>If yes, please specify which one …………………………………………………………………………..	☐ YES ☐ NO
A	2. Does the laboratory have an internal Ethical Committee?	☐ YES ☐ NO
D1/ A	3. Has the Director of the laboratory developed a statement or policy that confirms a commitment to risk management, assigning authority, responsibility, and accountability at the appropriate levels within the organization?	☐ YES ☐ NO
D3	4. Is the Risk Management System adequately taken into consideration when the laboratory processes, strategies, and activities are planned?	☐ YES ☐ NO
D1	5. Have the necessary resources for risk managing been adequately allocated?	☐ YES ☐ NO
D1	6. Is there a Risk Team in charge of identifying possible risks or adverse events that might occur evaluating them, during periodic meetings, in terms of occurring probability and severity and in charge of supervising the correct execution of the actions planned for mitigating them or, at least, to reduce the risk to a tolerable level?	☐ YES ☐ NO
D1/ A	7. Are a), b), and c) present in the laboratory?<br><br>a) Validated written instructions for each process, including management of adverse events<br>b) Warning and accident prevention signs used to minimize work hazards<br>c) Continuous training for laboratory biosafety and biosecurity practices	 <br>☐ YES ☐ NO<br>☐ YES ☐ NO<br>☐ YES ☐ NO
D1	8. Are adverse events, or incidents that might occur, rapidly communicated to the risk team?	☐ YES ☐ NO
D1	9. Are all the risks and actions to be taken for the risk management rapidly communicated to all the staff clearly, using plain language?	☐ YES ☐ NO

Ethics Laboratory for Veterinary Medicine, Conservation, and Animal Welfare, 2019. All rights reserved. Any use, in whole or in part, of the contents included in this document, including the storage, reproduction, reprocessing, dissemination or distribution of the contents through any technological platform, support or electronic network, without the prior written authorization of all the authors is prohibited.

D3	10. In case of emergencies, due to natural/human causes, has the laboratory developed and implemented plans describing the actions to be taken for the following aspects?	
	a) Safety of personnel	☐ YES ☐ NO
	b) Protection of all fresh and cryopreserved material	☐ YES ☐ NO
	c) Limitation of damage to equipment and medical records	☐ YES ☐ NO
D3/A	11. Is the access to the place where the biomaterials (biobank building, in the storage room, cryostorage units, *etc.*) are stocked appropriately controlled and documented?	☐ YES ☐ NO
D3/D2	12. Have equipment maintenance schedules, replacement timetables, and calibration (if needed), for ensuring proper and periodic maintenance, been planned?	☐ YES ☐ NO
D2	13. Are the following devices adequately monitored and equipped with alarm systems, for detecting any out of range temperature and/or liquid nitrogen low level?	
	a) Shipping incubator	☐ YES ☐ NO
	b) Cryo-storage units	☐ YES ☐ NO
C2	14. During the processing of specimens in arrival, are the following aspects been considered?	
	a) Unpackaging in biological safety cabinets	☐ YES ☐ NO
	b) Adequate label and register in a database	☐ YES ☐ NO
C2	15. Are sanitation and sterilization protocols applied for a), b), and c)?	
	a) Equipment	☐ YES ☐ NO
	b) Personnel	☐ YES ☐ NO
	c) Laboratories	☐ YES ☐ NO
D3	16. Is there a database that stores all the information related to the following aspects?	
	a) Gametes donors	☐ YES ☐ NO
	b) OPU/semen collection report	☐ YES ☐ NO
	c) Morphological characteristics of gametes/embryos	☐ YES ☐ NO
	d) Procedures/manipulation done on the gametes/embryos (including timing and staff involved)	☐ YES ☐ NO
	e) Documentation/records indicating the results of the embryos testing (including also the data of the embryos that failed to pass the quality test)	☐ YES ☐ NO

Ethics Laboratory for Veterinary Medicine, Conservation, and Animal Welfare, 2019. All rights reserved. Any use, in whole or in part, of the contents included in this document, including the storage, reproduction, reprocessing, dissemination or distribution of the contents through any technological platform, support or electronic network, without the prior written authorization of all the authors is prohibited.

C2	17. Are the following spermatozoa characteristics evaluated from the biobank before long-term stocking?	
	a) Concentration	☐ YES ☐ NO
	b) Motility	☐ YES ☐ NO
	c) Morphology	☐ YES ☐ NO
	d) Acrosome integrity	☐ YES ☐ NO
**PHASE B**	**GAMETES SHIPPING TO LABORATORY**	
A	18. Has a checklist of the documents (certifications, permits, authorization letters) required by the national and international authorities for specimens (semen and/or oocytes) shipping, been drawn up for a) and b)?	
	a) Export	☐ YES ☐ NO
	b) Import	☐ YES ☐ NO
D1	19. Have the training and certification of the person in charge of gametes (semen and/or oocytes) shipping been assessed?	☐ YES ☐ NO
C2	20. Is there a protocol to be applied in order to respond adequately to emergencies (e.g. temperature variations, such as spills, damages, or theft of materials during transportation and any other realistic and foreseeable emergencies)?	☐ YES ☐ NO
C2/ D2	21. Are the gametes transported into a portable device that can control the adequate temperature chain?	☐ YES ☐ NO
C2	22. Have methods to assess the specimens package condition before the sending and after the receiving been developed?	☐ YES ☐ NO
C2	23. Is it possible to track the shipment of the embryos in any phase?	☐ YES ☐ NO
C2	24. Are the gametes (sperm/oocytes) preserved from x-ray check control in the airports?	☐ YES ☐ NO
**PHASE C**	**GAMETES BIOBANKING**	
C2/ A	25. Are sample handling times documented?	☐ YES ☐ NO
C2	26. To avoid misidentification, are the specimens adequately labelled?	☐ YES ☐ NO
C2	27. Before stocking, are the specimens treated for preventing microbial contamination?	☐ YES ☐ NO
C2	28. Before stocking are the specimens analysed with quality tests?	☐ YES ☐ NO

Ethics Laboratory for Veterinary Medicine, Conservation, and Animal Welfare, 2019. All rights reserved. Any use, in whole or in part, of the contents included in this document, including the storage, reproduction, reprocessing, dissemination or distribution of the contents through any technological platform, support or electronic network, without the prior written authorization of all the authors is prohibited.

C2/A	29. Are all the Biobanks involved in the specimens stocking certified?	☐ YES ☐ NO
C3	30. Are the specimens of the same animal, if possible, separated after the collection and shipped to different Biobanks?	☐ YES ☐ NO
C3	31. In the Biobank, are the specimens of the same animal, if possible, separated and stored in a) and b)?	
	a) Different storage containers	☐ YES ☐ NO
	b) Different structures/rooms	☐ YES ☐ NO
**PHASE D**	**GAMETES PREPARATION FOR ICSI**	
**PHASE D1**	**OOCYTES MATURATION**	
C1	32. Is the oocytes maturation protocol, based on the present scientific knowledge, the best for this species?	☐ YES ☐ NO
C1	33. Is the protocol for oocytes maturation optimized for the species under the procedure?	☐ YES ☐ NO
C1	34. Is the protocol for oocytes maturation optimized on species related to the one under the procedure?	☐ YES ☐ NO
C1	35. Is the quality of oocytes, to be used in the procedure, evaluated with a standardized protocol and with an established grade of scoring?	☐ YES ☐ NO
C1	36. Are all the following aspects in oocytes maturation steps optimized for this species?	
	a) pH	☐ YES ☐ NO
	b) Temperatures	☐ YES ☐ NO
	c) Maturation timing	☐ YES ☐ NO
	d) Mediums	☐ YES ☐ NO
C3	37. If oocytes do not reach the metaphase II (stage suitable for ICSI), can they be used for another experiment and/or procedural optimization?	☐ YES ☐ NO
C2	38. Is the percentage of oocytes maturation consistent with the percentage of other related animal oocytes maturation reported in the scientific literature?	☐ YES ☐ NO
**PHASE D2**	**SPERM PREPARATION**	
C1	39. Has the semen conservation protocol been optimized on species under the procedure?	☐ YES ☐ NO
C1	40. Based on the present scientific knowledge, are the protocols for sperm cryogenic conservation and sperm collection protocol the best for the species under the procedure?	☐ YES ☐ NO

Ethics Laboratory for Veterinary Medicine, Conservation, and Animal Welfare, 2019. All rights reserved. Any use, in whole or in part, of the contents included in this document, including the storage, reproduction, reprocessing, dissemination or distribution of the contents through any technological platform, support or electronic network, without the prior written authorization of all the authors is prohibited.

C1	41. Has the quality of sperm, to be used in the procedure, been evaluated with a standardized protocol and with an established grade of scoring?	☐ YES ☐ NO
C1	42. Is the percentage of sperm recovery consistent with the percentage of other animal sperm recovery, as reported in the scientific literature?	☐ YES ☐ NO
C3	43. For each ICSI procedure, is it possible to thaw the minimum necessary aliquot of semen?	☐ YES ☐ NO
C3	44. Is it possible to use the spermatozoa not used in ICSI for other experiments and/or procedural optimization?	☐ YES ☐ NO
**PHASE E**	**INTRACYTOPLASMIC SPERM INJECTION (ICSI)**	
C3	45. Is the piezo-driven micromanipulator used the best available that can avoid oocyte damage and improve the fertilization rate? (e.g. a piezo-stepper that can control the pressure of the intracytoplasmatic injection)	☐ YES ☐ NO
C3	46. Is ICSI the best fertilization practice with the highest percentage of egg fertilization success?	☐ YES ☐ NO
C1	47. In order to improve fertilization rate, is the maturation time of the oocytes used optimized?	☐ YES ☐ NO
C1	48. In order to prevent oocyte damage, are all the steps of oocytes manipulation (cumulus cell removal, sperm injection position in the membrane, etc.) optimized?	☐ YES ☐ NO
C1	49. In order to prevent oocyte damage, are hyaluronidase concentration and exposure timing optimized to be kept to a minimum to remove cumulus cells from oocytes?	☐ YES ☐ NO
C1	50. Is there a technique to improve the low rate of fertilization and cleavage after sperm injection?	☐ YES ☐ NO
**PHASE F**	**EMBRYOS CULTURE**	
C1	51. Is the embryo culture protocol the best available, based on present scientific knowledge, for this species?	☐ YES ☐ NO
C1	52. Is the embryo culture protocol optimized on species under the procedure?	☐ YES ☐ NO
C1	53. Is the embryo culture protocol optimized on species related to the one under the procedure?	☐ YES ☐ NO
C3	54. Is the timing of medium changing optimized for this species?	☐ YES ☐ NO
C1	55. Are all the data related to the status of the embryo adequately recorded?	☐ YES ☐ NO

Ethics Laboratory for Veterinary Medicine, Conservation, and Animal Welfare, 2019. All rights reserved. Any use, in whole or in part, of the contents included in this document, including the storage, reproduction, reprocessing, dissemination or distribution of the contents through any technological platform, support or electronic network, without the prior written authorization of all the authors is prohibited.

C1/ D3	56. Do all the manipulation of the embryos take place in an adequate place (in terms sanification, cleanliness etc.) with sanitized materials?	☐ YES ☐ NO
C1	57. Is the quality of embryos evaluated, before cryopreservation, with a standardized protocol and with an established grade of embryo scoring?	☐ YES ☐ NO
**PHASE G**	**EMBRYOS CRYOPRESERVATION AND BIOBANKING**	
D3	58. Is there a database including the following information?	
	a) Cryogenic vial label information (i.d. Number, *etc.*)	☐ YES ☐ NO
	b) Cryopreservation method	☐ YES ☐ NO
	c) Date and time of cryopreservation	☐ YES ☐ NO
	d) Operator	☐ YES ☐ NO
	e) Parents' information	☐ YES ☐ NO
	f) Embryo quality and stage of development	☐ YES ☐ NO
	g) Number of embryos per cryogenic vial	☐ YES ☐ NO
	h) Location (tank, canister) of stored samples in the cryogenic device	☐ YES ☐ NO
D3	59. Is there a periodic inventory of the contents of the cryobank, including cross-referencing contents with storage records?	☐ YES ☐ NO
C3	60. Is the slow freezing protocol used for embryo cryopreservation the best available for the species?	☐ YES ☐ NO
C3	61. Would it be possible to have a higher rate of embryos recovery with the vitrification protocol?	☐ YES ☐ NO
**PHASE H**	**EMBRYOS PACKAGING**	
A	62. To carry out the process in line with the relevant national and international regulations, has the staff involved in the embryos packaging been adequately assessed regarding the following aspects?	
	a) Training	☐ YES ☐ NO
	b) Certification	☐ YES ☐ NO
	c) Competence	☐ YES ☐ NO
C1	63. Is the embryos packaging adequate in terms of a) and b)?	
	a) Temperature control	☐ YES ☐ NO
	b) Incubator volume limit	☐ YES ☐ NO
C1	64. Is the marking and labelling for the identification and classification of the packed biological material adequate?	☐ YES ☐ NO
C1	65. Have different tools (e.g. checklist, etc) and/or procedure been developed to check the correct packaging?	☐ YES ☐ NO

Ethics Laboratory for Veterinary Medicine, Conservation, and Animal Welfare, 2019. All rights reserved. Any use, in whole or in part, of the contents included in this document, including the storage, reproduction, reprocessing, dissemination or distribution of the contents through any technological platform, support or electronic network, without the prior written authorization of all the authors is prohibited.

PHASE I	EMBRYOS SHIPPING	
A	66. Has a checklist to verify documents (certifications, permits, authorization letters) required by the national and international authorities for specimens (gametes) shipping, been drawn up for the following details?<br>a) Export<br>b) Import	 ☐ YES ☐ NO<br>☐ YES ☐ NO
A	67. Have the training and certification of the person in charge of embryo shipping been assessed?	☐ YES ☐ NO
C3	68. Is there a protocol to be applied in order to respond adequately to emergencies (e.g. temperature variations, such as spills, damages, or theft of materials during transportation and any other realistic and foreseeable emergencies)?	☐ YES ☐ NO
D2	69. Are the embryos transported into a portable device that can control the adequate temperature chain?	☐ YES ☐ NO
C1	70. Have methods to assess the specimens package condition before the sending and after the receiving been developed?	☐ YES ☐ NO
C1	71. Is it possible to track the shipment of the embryos in any phase?	☐ YES ☐ NO
C2	72. Are the embryos preserved from x-ray check control in the airports?	☐ YES ☐ NO

Ethics Laboratory for Veterinary Medicine, Conservation, and Animal Welfare, 2019. All rights reserved. Any use, in whole or in part, of the contents included in this document, including the storage, reproduction, reprocessing, dissemination or distribution of the contents through any technological platform, support or electronic network, without the prior written authorization of all the authors is prohibited.

Comments,

I hereby give my consent for the processing of data provided on the Ethical Risk Assessment (ERA) form to be stored, processed, analyzed and published by B*io*R*escue* project partner for scientific research purposes.

Place, Date

Signature

Ethics Laboratory for Veterinary Medicine, Conservation, and Animal Welfare, 2019. All rights reserved. Any use, in whole or in part, of the contents included in this document, including the storage, reproduction, reprocessing, dissemination or distribution of the contents through any technological platform, support or electronic network, without the prior written authorization of all the authors is prohibited.
